# EnteroBase: hierarchical clustering of 100 000s of bacterial genomes into species/subspecies and populations

**DOI:** 10.1098/rstb.2021.0240

**Published:** 2022-10-10

**Authors:** Mark Achtman, Zhemin Zhou, Jane Charlesworth, Laura Baxter

**Affiliations:** University of Warwick, Coventry CV4 7AL, UK

**Keywords:** big data, hierarchical clustering, genomic databases, EnteroBase, cgMLST, accessory genome

## Abstract

The definition of bacterial species is traditionally a taxonomic issue while bacterial populations are identified by population genetics. These assignments are species specific, and depend on the practitioner. Legacy multilocus sequence typing is commonly used to identify sequence types (STs) and clusters (ST Complexes). However, these approaches are not adequate for the millions of genomic sequences from bacterial pathogens that have been generated since 2012. EnteroBase (http://enterobase.warwick.ac.uk) automatically clusters core genome MLST allelic profiles into hierarchical clusters (HierCC) after assembling annotated draft genomes from short-read sequences. HierCC clusters span core sequence diversity from the species level down to individual transmission chains. Here we evaluate HierCC's ability to correctly assign 100 000s of genomes to the species/subspecies and population levels for *Salmonella, Escherichia, Clostridoides, Yersinia, Vibrio* and *Streptococcus*. HierCC assignments were more consistent with maximum-likelihood super-trees of core SNPs or presence/absence of accessory genes than classical taxonomic assignments or 95% ANI. However, neither HierCC nor ANI were uniformly consistent with classical taxonomy of *Streptococcus.* HierCC was also consistent with legacy eBGs/ST Complexes in *Salmonella* or *Escherichia* and with O serogroups in *Salmonella*. Thus, EnteroBase HierCC supports the automated identification of and assignment to species/subspecies and populations for multiple genera.

This article is part of a discussion meeting issue ‘Genomic population structures of microbial pathogens’.

## 1. Introduction

Microbiologists need large databases to identify and communicate about clusters of related bacteria, … Such databases should contain the reconstructed genomes of bacterial isolates, … together with metadata describing their sources and phenotypic properties … and we are currently developing EnteroBase, a genome-based successor to the *E. coli* and *S. enterica* MLST databases.

Achtman and Zhou, 2014 [1].

The Linnean system of genus and species designations was applied to bacterial nomenclature in the late ninteenth century, soon after their ability to cause infectious diseases had been recognized. These taxonomic labels initially reflected disease specificity and phenotypic similarities. Over the following decades, phenotypic criteria for distinguishing bacterial taxa were extended to include serologically distinct groupings and even differential sensitivity to bacteriophages. A primary goal for taxonomic designations for bacterial pathogens was that they be useful for epidemiology and clinical diagnoses. As a result, serovars of *Salmonella enterica*, which differ by dominant epitopes on lipopolysaccharide and flagella, were each assigned a distinct species designation, often referring to the disease syndrome and host, e.g. *Salmonella typhimurium* [2]. Similarly, even though *Yersinia pestis* is a clone of *Yersinia pseudotuberculosis* [3,4], it was designated as a distinct species because *Y. pestis* causes plague whereas *Y. pseudotuberculosis* causes gastroenteritis. The use of species designations for *Salmonella* serovars is now disparaged, although it is still in common use [5], but *Y. pestis* has retained its species designation.

Percentage DNA–DNA hybridization levels became the ‘Gold Standard’ metric for new taxonomic designations after 1987 [6]. DNA-DNA hybridization reflects genomic relationships but distinctive phenotypic differences have remained a requirement for the definition of a novel species until the present. Indeed, the international committee which controls taxonomic designations continues to reject the validity of taxonomic designations based solely on DNA similarities [7,8].

### (a) Average nucleotide identity and multilocus sequence typing

An alternative genomic approach for taxonomic designations was proposed in 2005 by Konstantinidis [9], namely the definition of species on the basis of average nucleotide identity (ANI). Pairs of genomes with 95% ANI usually belong to the same species whereas pairs with lower ANI values belong to different species. Computational methods based on k-mer searches, such as FastANI [10], can rapidly perform ANI-like calculations on large numbers of genomes, and these calculations are now routinely used by bioinformaticians for taxonomic assignments. An approach based on ANI is enticing because it could provide defined criteria for the definition of a species across all Bacteria, and allow species assignment based exclusively on genome sequences. However, the use of ANI for species assignments has been criticized because it does not completely correlate with DNA-DNA hybridization [11,12]. Furthermore, multiple taxa that are designated as single species each contain multiple 95% ANI groups [13,14]. For example, strains of *Streptococcus mitis* colonize the human oropharyngeal tract, have similar phenotypes and are considered to represent a single species. However, *S. mitis* encompasses myriad, genetically distinct strains [15–17], and encompasses at least 44 distinct 95% ANI clusters [17]. Other problematic genera include *Pseudomonas* [12,18] and *Aeromonas* [19]. Furthermore, large databases including 100 000s of genomes per genus would struggle to implement methods such as pairwise clustering by ANI because each new entry would require testing against all genomes. There is also no consensus on ANI criteria for recognizing lower taxonomic entities, such as subspecies and populations. Indeed, there is not even a consensus on the definitive properties of what constitutes a bacterial species [20].

Bottom-up population genetic approaches such as multilocus sequence typing (MLST) can provide an alternative to top-down taxonomy, and deal efficiently with large numbers of bacterial strains. Legacy MLST based on the sequence differences of several housekeeping genes was introduced in 1998 [21], and has now been applied to more than 100 bacterial species [22]. MLST defines sequence types (STs), consisting of unique integer designations for each unique sequence (allele) of each of the MLST loci. Some STs mark individual clones with special pathogenic properties and which seem to have arisen fairly recently, such as *Escherichia coli* ST131, which is a globally prominent cause of urinary tract infection (UTI) and invasive disease [23]. Similarly, *Salmonella enterica* subsp. *enterica* serovar Typhimurium ST313 is a common cause of extra-intestinal, invasive salmonellosis in Africa [24]. Higher order clusters of related STs are also well known. Such clusters can be recognized by eBurst analyses [25], and are referred to as ST Complexes in *E. coli* [26] and eBGs (eBurst groups) in *S. enterica* [27]. ST Complexes and eBGs seem to reflect natural populations, but their broad properties are still not well understood [28].

The principles of legacy MLST were extended to rMLST, which uses sequences of 53 universal bacterial genes encoding ribosomal proteins and provides a universal MLST scheme for all Bacteria [29]. For both *Escherichia* and *Salmonella,* rMLST offers a slight improvement in resolution over legacy MLST, and the identification of *Salmonella* eBGs is reasonably consistent across both approaches [30]. rMLST has been used to identify tractable sets of representative genomes from large collections for calculating phylogenetic trees [28] and pan-genomes [17].

MLST has also been extended to cgMLST, which encompasses all the genes in a soft core genome [29–31], and cgMLST nomenclatures have been implemented for multiple bacterial genera [28]. The large number of loci encompassed by cgMLST schemes results in enormous numbers of cgMLST STs (cgSTs), but these can be clustered into groups of bacterial genomes at multiple levels of genomic diversity (hierarchical clustering) [32]. cgMLST provides considerably higher resolution than legacy MLST or rMLST, and initial analyses indicated that it is ideal for investigating transmission chains within single source outbreaks or for identifying population structures up to the genus level [28]. Here we focus on the automated assignments of genome assemblies from six genera of important bacterial pathogens to taxonomic and population structures by hierarchical clustering of cgSTs with the EnteroBase HierCC pipeline (table 1) [32]. For five of those genera, HierCC is a full solution for taxonomic designations of species.

**Table 1.** Legacy data, newly assembled genomes and hierarchical cgST clusters in EnteroBase (09/2021). NOTE: no. genomic loci is numbers of coding sequences whose alleles are automatically called for the wgMLST and cgMLST schemes; HierCC level: maximum numbers of allelic differences within minimal spanning trees that define HC clusters of cgSTs. EnteroBase automatically calculates existing Legacy MLST STs according to legacy schemes for *Escherichia/Shigella* (Wirth *et al.* [26]), *Salmonella* (Achtman *et al.* [27]), and *Clostridioides* (Griffiths *et al.* [33]), but does not assign new STs nor does it maintain a database of legacy data from ABI sequencing. Additional public databases are presented by EnteroBase for *Helicobacter* (>3500 genomes) and *Moraxella* (>2350 genomes), but these lack a cgMLST scheme. EnteroBase also has a database for >80 000 *Mycobacterium* genomes, but this currently (March, 2022) lacks a cgMLST scheme.

genus	legacy MLST (no. strains)	cgMLST (no. genomes)	no. genomic loci		no. HierCC clusters (HierCC level)		
			wgMLST	cgMLST	ST complexes	Lineages	species/subspecies
*Salmonella*	4930	312 196	21 065	3002	3648 (900)	2185 (2000)	15 (2850)
*Escherichia/Shigella*	9525	175 256	25 002	2513	1379 (1100)	343 (2000)	16 (2350)
*Streptocococcus*		76 718	33 887	372	3681 (100)		132 (363)
*Clostridioides*		23 197	11 490	2556	299 (950)		12 (2500)
*Vibrio*		12 267	152 249	1128	2000 (800)		155 (1090)
*Yersinia*	4286	4023	19 591	1553	451 (600)		34 (1490)

### (b) EnteroBase and HierCC

EnteroBase (http://enterobase.warwick.ac.uk) includes tools for automatic downloading of short-read sequences and their metadata from the public domain, assembly into draft genomes and population genetic analyses of the core and accessory genomes (table 2) [28]. Draft genomes are annotated according to genus-wide pan-genome schemes created with PEPPAN [17]. In September 2021, EnteroBase contained over 600 000 draft genomes from the six genera in table 1, and likely provides a nearly comprehensive overview of their global diversity. Many of these samples reflect a focus on food-borne disease in the US and United Kingdom, but this bias is increasingly being reduced by the global sources of many genomes (see electronic supplementary material, text, Sample Bias).

**Table 2.** EnteroBase-related software tools that support the analysis of large numbers of bacterial genomes. NOTE: PEPPAN is a stand-alone program that was used to generate the wgMLST schemes used in EnteroBase. SPARSE is a second stand-alone program that has been used to extract taxon-specific reads from metagenomes of ancient DNA that were then used with EToKi to define pseudo-MAGs (metagenomic assembled genomes) that were uploaded to EnteroBase for phylogenetic comparisons. HierCC was used as a stand-alone program in development mode to define an initial set of clusters from a representative set of genomes. Subsequent assignments to existing clusters or to novel clusters were performed automatically within EnteroBase using pHierCC in production mode. BlastFrost is used by EnteroBase to determine the presence/absence of toxins and other pathovar characteristics of gastrointestinal pathogens within *E. coli*, and to assign gastrointestinal pathovar designations.

name	purpose	citation	URL for stand-alone version
GrapeTree	GUI for depicting and analysing minimal spanning and NJ trees of character data	[34]	https://github.com/achtman-lab/GrapeTree
PEPPAN	pan-genome calculation including pseudo-genes from numerous representatives of an entire genus	[17]	https://github.com/zheminzhou/PEPPAN
SPARSE	assignment of metagenomic reads to individual taxa	[35,36]	https://github.com/zheminzhou/SPARSE
EToKi	EnteroBase toolkit of useful functions and pipelines	[28]	https://github.com/zheminzhou/EToKi
BlastFrost	efficient k-mer based search for DNA sequences in large genomic datasets	[37]	https://github.com/nluhmann/BlastFrost
HierCC	automated hierarchical clustering of cgSTs to existing or novel clusters	[32]	https://github.com/zheminzhou/pHierCC

One of the primary goals for EnteroBase was a hierarchical overview of the population structure of the genera in table 1. We therefore developed HierCC [32] based on cgMLST assignments to support the rapid recognition and detailed investigation of differing levels of population structure. EnteroBase reports cluster assignments and designations at 10–13 levels of allelic differences for all six genera [28] (table 1). HC5–HC10 clusters with maximal internal pair-wise distances within minimal spanning trees of 5 or 10 alleles, respectively, have been used to identify short-term, single source outbreaks of *S. enterica* and *E. coli/Shigella* that extended to multiple European countries [38–42]. Pathogen species can also include higher level clusters, which can correspond to somewhat more distantly related bacterial populations that cause endemic or epidemic disease over longer time periods in one or more countries [43–46]. EnteroBase HierCC has even been used to classify all *Shigella* [47], which correspond to discrete lineages of *E. coli* [26,48,49].

Classical taxonomic approaches for assigning individual strains and genomes to species depend on human expertise and are not suitable for automated pipelines in real-time databases such as EnteroBase. This problem is acute because many short read sequences in the European Nucleotide Archive (ENA) have incorrect taxonomic assignments, or none at all, and phenotypic distinctions are inappropriate for databases containing 100 000s of genomes. Zhou *et al.* compared taxonomic designations with peak normalized mutual information and silhouette scores for HierCC clusters and identified HierCC levels that corresponded with well-defined species/subspecies in each genus [32]. For example, for *Salmonella* with a total of 3002 loci in the cgMLST scheme, HC2850 (94.9% of all cgMLST alleles) was chosen as the optimal HC level for identifying species and subspecies. The optimal HC levels for the other five genera with cgMLST schemes ranged from HC363 to HC2500 (93.6–97.8%) (table 1). Here we compare the consistency of those HierCC assignments with assignments based on classical taxonomic approaches and 95% ANI. The results illustrate problems with classical approaches and with 95% ANI, and we conclude that HierCC is preferable for automated assignment of genomes to species/subspecies for those genera. However, neither ANI nor HierCC is universally satisfactory for species/subspecies assignments within *Streptococcus.* This manuscript also provides an initial overview of the abilities of HierCC to assign genomes to populations and Lineages [50,51] within *Salmonella* and *Escherichia*/*Shigella*, and compares those assignments with the distributions of O antigens within lipopolysaccharide.

## 2. Results

### (a) Species and subspecies

In order to test the efficacy of HierCC at identifying species and subspecies, we extracted collections of representative genomes from all six genera in EnteroBase for which HierCC clusters had been implemented (table 3). We wished to calculate maximum-likelihood (ML) phylogenetic topologies of these genomes based on their core genome single nucleotide polymorphisms (SNPs) or the presence/absence of accessory genes from the pan-genome. Such ML trees are very slow to calculate with large datasets, but disjointed tree merging (DTM) within a divide-and-conquer approach allows large phylogenetic trees to be calculated in a reasonable time [52]. We therefore developed cgMLSA (see electronic supplementary material, Methods in Supplementary Text), a novel DTM approach based on ASTRID [53] and ASTRAL [54] that enabled the calculations of ML super-trees from up to 10 000 representative genomes within hours to days, and applied it to each of the datasets. The resulting ML super-trees were annotated with taxonomic designations and cluster designations according to a 95% ANI cutoff calculated with FastANI [10] (95% ANI clusters). We then compared those annotations with cluster assignments based on the species-specific HierCC levels in table 1.

**Table 3.** Parameters of datasets used for calculating ML super-trees. Each dataset consists of genomes representing the entire diversity of a genus as described in Methods (see electronic supplementary material, Supplemental Text).

genus	no. type strains	total no. genomes	no. strict core genes	no. accessory genes	no. soft core genes (cgMLST)	no. SNPs
*Salmonella*	7	10 002	1409	19 486	3002	1 410 331
*Escherichia* (EcoRPlus)	8	9479	134	20 294	2513	1 172 200
*Escherichia* reps	2	967	192	17 983	2513	971 516
*Streptocococcus*	84	5937	90	31 630	372	263 080
*Clostridioides*	1	6725	515	10 975	2556	725 240
*Vibrio*	119	5032	124	146 041	1128	776 439
*Yersinia*	23	1847	744	18 799	1553	656 143

#### (i) Salmonella

**Figure 1.**
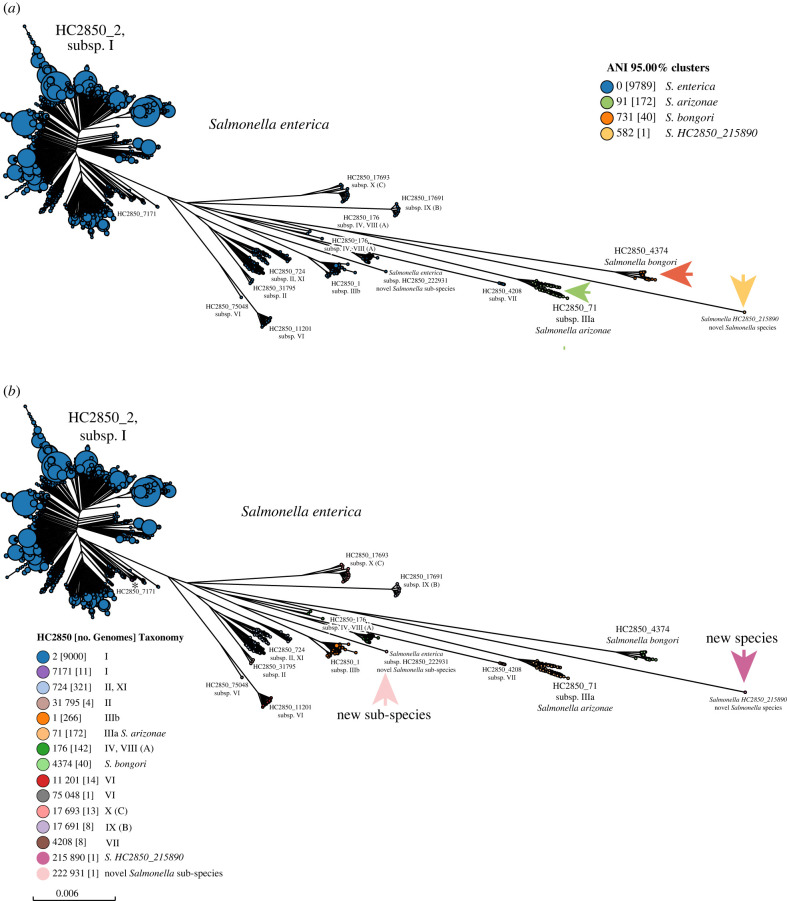
A comparison of species and subspecies assignments within *Salmonella* with HierCC and ANI. The figure shows an ML super-tree of 1 410 331 SNPs among 3002 core genes from 10 002 representative genomes of *Salmonella* (table 3). Former subspecies IIIa is designated *S. arizonae* in accordance with Pearce *et al.* 2021 [55]. (*a*) Partitions differentiated by ANI 95% clusters (legend) correspond to species *S. enterica, S. bongori*, *S. arizonae* and a new species, *S. HC2850_215890* (five strains from the UK, 2018–2020), as indicated by arrows, and subspecies are not differentiated. (*b*) Partitions coloured by HC2850 clusters (legend). Arrows indicate HC2850_215890, and a new subspecies, HC2850_222931 (one strain from France, 2018). All other HC2850 clusters correspond to species (*S. bongori* and *S. arizonae*) or subspecies, except for HC2850_7171 (starred), which is subsp. *enterica* (I) according to the ML tree. An interactive version of this GrapeTree rendition can be found at https://enterobase.warwick.ac.uk/ms_tree?tree_id=53257. The corresponding presence/absence tree can be found at https://enterobase.warwick.ac.uk/ms_tree?tree_id=53258.

The topologies of ML super-trees based on 1 410 331 core SNPs from 10 002 representative genomes were in large part concordant with traditional taxonomic assignments, and with clustering according to 95% ANI (figure 1*a*) or HC2850 (figure 1*b*). The three sets of assignments were also largely concordant with the topology of an ML super-tree based on the presence or absence of accessory genes (https://enterobase.warwick.ac.uk/ms_tree/53258). HC2850 and 95% ANI distinguished *S. enterica* from *S. bongori*, and from former subspecies IIIa, which was recently designated *S. arizonae* by Pearce *et al*. [55]. HC2850 also identified another new *Salmonella* species, cluster HC2850_215890, and that identification was confirmed by 95% ANI. HC2850_215890 consists of five strains that have been isolated from humans since 2018 in the UK, and a gene for gene comparison indicated that roughly half of their core genes were more similar to *S. enterica* and the other half to *S. bongori*.

Ninety-five percent ANI did not detect any subspecies structure within *S. enterica* whereas HC2850 differentiated a total of 12 clusters that corresponded to distinct phylogenetic branches in the ML super-tree (figure 1*b*). Most of these have been designated as subspecies by traditional taxonomy [30,55], except for a singleton genome in HC2850_222931, which likely represents a novel subspecies. Despite the correct identification of so many subspecies, HC2850 did not support Pearce's definition of subspecies XI and did not distinguish those genomes from subsp. II. Similarly, HC2850 did not differentiate Pearce's subsp. VIII from subsp. IV. The prior definition of these subspecies was largely dependent on branch topologies within phylogenetic trees based on rMLST loci, and was also contradicted by population statistics [55].

HierCC also differentiated HC2850_7171, which consists of a short phylogenetic branch within subsp. I that would not have been assigned to a subspecies according to its ML topology. Genomic comparisons of multiple single genes from genomes within HC2850_7171 suggest that HC2850_7171 may be a hybrid between subsp. I and II because some gene sequences were most similar to the former and others were most similar to the latter. With this sole possible exception, HierCC seems to be suitable for the automated detection of new *Salmonella* species and subspecies, and for routinely assigning novel genomes to the appropriate taxa.

#### (ii) Escherichia

In addition to *Escherichia coli, Escherichia* includes the named species *albertii* [56–58], *fergusonii* [59,60]*, marmotae* [61,62] and *ruysiae* [63]. And despite their apparently distinct genus and species names, *Shigella boydii, Shigella dysenteriae, Shigella flexneri* and *Shigella sonnei,* all common causes of dysentery, correspond to phylogenetic lineages within *E. coli* [48] rather than to discrete taxonomic units. Still other, unusual *Escherichia* strains from lake and ocean water are associated with long phylogenetic branches, and were designated as **‘**cryptic clades’ I–VIII by population geneticists [64–69]. The branch leading to clade I is simply a long phylogenetic branch within *E. coli* [64]. However*,* clade V encompasses *E. marmotae* [61,62] and *E*. *ruysiae* consists of the union of clades III and IV [63]. Initial analyses with the EcoRPlus collection of 9479 genomes [28] (table 3) yielded results that were compatible with these interpretations. Almost all *E. coli* or *Shigella* genomes were within HierCC cluster HC2350_1, and genomes with other taxonomic or clade designations belonged to other HC2350 clusters. However, soon after the definition of the EcoRPlus collection, additional genomes of *Escherichia* which were related to clade II were described from inter-tidal marine and fresh-water sediments near Hong Kong [66,70]. Furthermore, due to its numerical predominance, *E. coli* overshadows other *Escherichia* species and subspecies within EcoRPlus*.* We therefore, created *Escherichia* reps in Jan 2021, a novel set of 967 representative genomes which included one genome from each of the 160 most common HC1100 clusters in HC2350_1 and all 807 genomes from other HC2350 clusters which existed in EnteroBase at that time.

**Figure 2.**
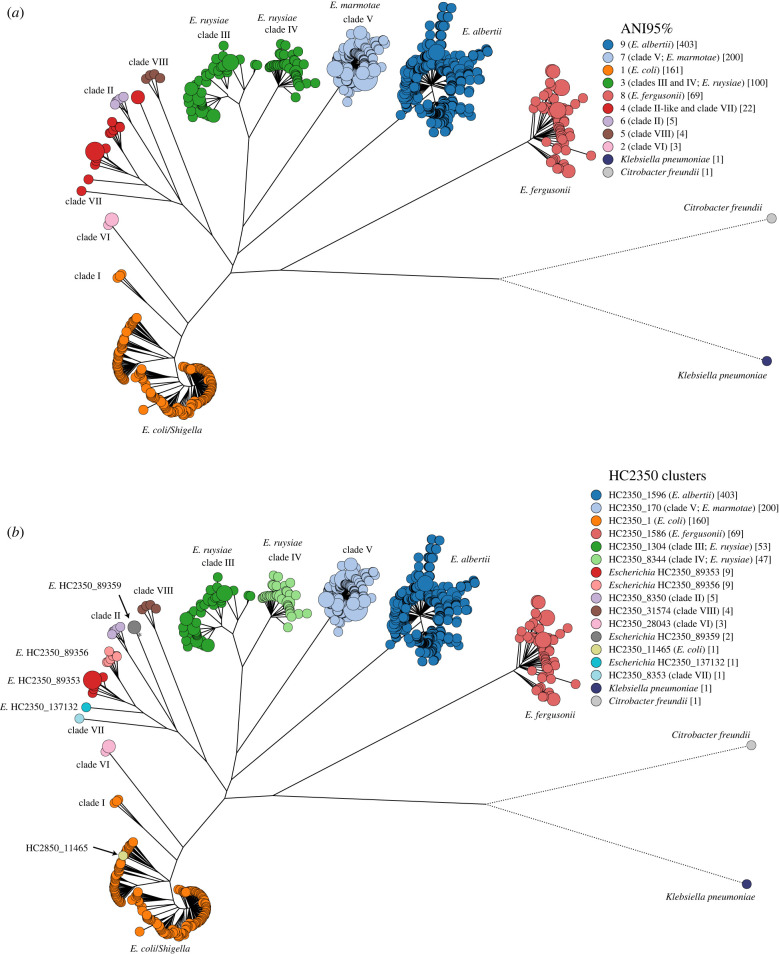
Maximum-likelihood core SNP tree of 967 *Escherichia* genomes consisting of one genome from each of 161 HC1100 clusters containing *E. coli* or *Shigella* as well as all 806 other *Escherichia* genomes in EnteroBase as of November 2020. The tree is coloured by (*a*) pairwise FastANI values clustered at the 95% level and (*b*) HC2350 cluster designations. The key legends indicate taxonomic designations in the literature which best match the cluster groupings. In (*b*), HC2350 cluster designations were used to mark novel taxonomic groupings in HC2350 clusters 89353, 89356, 89359 and 137132. An interactive version of this GrapeTree rendition can be found at https://enterobase.warwick.ac.uk/ms_tree?tree_id=52101. The corresponding presence/absence tree can be found at https://enterobase.warwick.ac.uk/ms_tree?tree_id=71125.

Figure 2 shows that *E. fergusonii* and *E. albertii* form discrete 95% ANI clusters as do *E. marmotae* and other clade V genomes. As previously reported [63], *E. ruysiae* consists of the distinct clusters of clades III and IV. Three distinct 95% ANI clusters were found within clades II, VI and VIII while the Hong Kong genomes represented multiple, related phylogenetic branches, one of which has previously been designated clade VII. All *E. coli* and *Shigella* genomes are in a common 95% ANI group, as is clade I. Comparable clustering results were also found in an ML tree of presence/absence of accessory genes, albeit with different topology of the deepest branches resulting in *E. fergusonii* and clade VIII being most closely related to *E. coli* (https://enterobase.warwick.ac.uk/ms_tree?tree_id=71125). These observations indicate that the taxonomy and population genetic designations for *Escherichia* are incomplete, and also partially inconsistent.

HierCC clustering of the same data provided a consistent and uniform nomenclature. Each discrete phylogenetic cluster received its own HC2850 designation, including clades III and IV and four phylogenetic clusters among the Hong Kong isolates which were designated according to their HC cluster number: e.g. *Escherichia* HC2350_89353. *E. coli, Shigella* and clade I all clustered together in HC2350_1, and the only discordance between topological clustering and HierCC clustering among the 967 genomes in *Escherichia* reps was a single genome which belonged to *E. coli* by phylogenetic topology but was assigned to HC2350_11465 by HierCC. These results were so convincing that HierCC groupings were used in late 2021 to curate and update all species designations for *Escherichia* entries within EnteroBase.

#### (iii) Clostridioides

**Figure 3.**
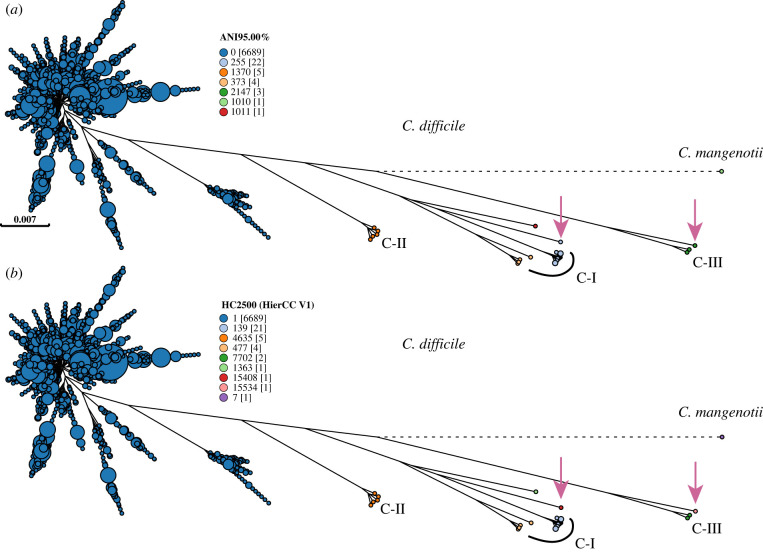
Species and subspecies assignments within *Clostridioides* according to 95% ANI (*a*) and HierCC (*b*). ML super-tree of 725 240 SNPs among 2556 core genes from 6724 representative genomes of *Clostridioides difficile* and one genome of *Clostridioides mangenotii* (table 3)*.* (*a*) ANI 95% clusters differentiate *C. mangenotii* (cluster 1010) and five other clusters (clusters 255, 1370, 373, 2147, 1011) from *C. difficile* (cluster 0). Four of the 95% ANI clusters correspond to cryptic clades C-I (clusters 255, 373), C-II (cluster 1370) and C-III (cluster 2147) in the designations by Knight *et al.* [71]. Arrows indicate two additional clusters that were distinguished by HierCC in (*b*). (*b*) Partitions coloured by HierCC assign the same genomes to HC2500 clusters as ANI, except that HierCC assigns HC2500_15334 and HC2500_15408 designations to one genome each. An interactive version of this GrapeTree rendition can be found at https://enterobase.warwick.ac.uk/ms_tree?tree_id=53253 and a presence/absence tree can be found at https://enterobase.warwick.ac.uk/ms_tree?tree_id=53254.

*Clostridium difficile* clustered distinctly from *C. mangenotii* according to 95% ANI*,* and ANI also distinguished five other clusters among genomes that were designated *C. difficile.* These clusters seem to be novel species according to their phylogenetic topology in the ML SNP super-tree (figure 3). HC2500 identified the same clusters, and also separated out two additional distinct clusters which resemble novel subspecies (arrows: HC2500_15334, HC2500_15408). A subset of the genomes in these clusters (figure 3) were assigned to cryptic clades C-I, CII and C-III by a recent publication [71], which also concluded that they represent novel species. Thus, HierCC is also suitable for the automated detection of new *Clostridioides* species and subspecies, and for routinely assigning novel genomes to the appropriate taxa.

#### (iv) *Yersinia* and *Vibrio*

Detailed results with *Yersinia* and *Vibrio* can be found in the electronic supplementary material, text. The general take-home lessons from those analyses resemble those summarized above: 95% ANI clustering is largely congruent with existing taxonomic designations, and HierCC identifies both species and subspecies with better resolution than 95% ANI. However, multiple, unanticipated taxonomic problems were evident in both genera. Firstly, ENA uses the strain designation as a species designation when a species is not specified during the submission process. The resulting public metadata is bloated with inaccurate species names. Secondly, multiple distinct phylogenetic branches were found without any species identifier. Thirdly, in multiple cases the species identifier did not correspond with ANI and/or HierCC, and comparisons with both the SNP and presence/absence ML topologies confirmed that the species designations in ENA were incorrect. We addressed these problems within EnteroBase by performing radical manual curation to the metadata to ensure that the taxonomic species designations correspond to the phylogenies and population structures. We also encountered multiple, problematical taxonomic designations which are described in the next paragraphs.

#### (v) Yersinia

Ninety-five percent ANI supports the taxonomic convention that *Y. enterocolitica* represents a single species, and all genomes designated as *Y. enterocolitica* were in a single 95% ANI cluster. However, in accord with the conclusions by Reuter *et al.* [72], HierCC clustering assigned the non-pathogenic biotype 1A genomes to HC1490_73 and HC1490_764, the highly pathogenic biotype 1B to HC1490_2, and pathogenic biotypes 2–5 to HC1490_10. We therefore renamed these four groups by adding the HierCC cluster to the species name, e.g. *Y. enterocolitica* HC1490_2.

In contrast to *Y. enterocolitica,* the *Yersinia pseudotuberculosis* Complex corresponds to a single phylogenetic cluster according to both HC1490 and 95% ANI (electronic supplementary material, figure S1). Taxonomists have split these bacteria into *Y. pseudotuberculosis*, *Y. pestis, Y. similis* and *Y. wautersii* [73,74]. DNA–DNA hybridization [75] and gene sequences of several housekeeping genes [4] previously demonstrated that *Y. pestis* is a clade of *Y. pseudotuberculosis,* and the new observations demonstrate that a distinct species status is not consistent with the genomic data for *Y. similis* and *Y. wautersii*. We have therefore downgraded all three taxa within EnteroBase to the category of subspecies of *Y. pseudotuberculosis,* and extended the designation of *Y. pseudotuberculosis* to *Y. pseudotuberculosis sensu stricto.* These assignments are not reflected by distinct HC1490 clusters, and HC1490 clustering can only be used to automatically assign new genomes to the *Y. pseudotuberculosis* Complex.

We also defined eight other novel species/subspecies designations within *Yersinia* and assigned unique designations based on HierCC clusters: e.g. *Yersinia* HC1490_419. These clusters were previously unnamed, or had been incorrectly designated with the names of other species which formed distinct ANI and HC1490 clusters (electronic supplementary material, text, figure S1).

#### (vi) Vibrio

*Vibrio* encompassed 152 HC1090 clusters (electronic supplementary material, figure S2, text), which corresponds to much greater taxonomic diversity than for the other genera dealt with above. The concordance between HC1090 and 95% ANI clusters was absolute for most clusters, including a large numbers of genomes from *V. cholerae, V. parahaemolyticus*, *V. vulnificans* and *V. anguillarum* (electronic supplementary material, table S1), and did not support the existence of additional subspecies. However, eleven HC1090 clusters each encompassed between two and five ANI clusters (electronic supplementary material, table S2) and three ANI clusters each encompassed 2–3 HC1090 clusters (electronic supplementary material, table S3). In order to support the automated assignment of genomes to named species, we implemented taxonomic assignments according to HC1090 and renamed the species of all genomes that were contradictory to this principle. The resulting dataset contains 109 HC1090 clusters with a unique, classical species designation as well as 43 other species level clusters designated as *Vibrio* HC1090_xxxx. Seventeen species names were eliminated because they were contradictory to the phylogenetic topologies or were incoherently applied (electronic supplementary material, text, table S4). These taxonomic changes now permit future automated assignment of novel genomes to species designations, and the recognition of novel species, and have provided a clean and consistent basic taxonomy that can be progressively expanded.

Prior work has assigned many *Vibrio* species into so-called higher order clades of species on the basis of MLSA (multilocus sequence analysis) [76]. These clades are also apparent in the ML super-tree of core SNPs, and electronic supplementary material, figure S2 indicates the three largest (Cholerae, Harveyii, Splendidus).

#### (vii) Streptococcus

**Figure 4.**
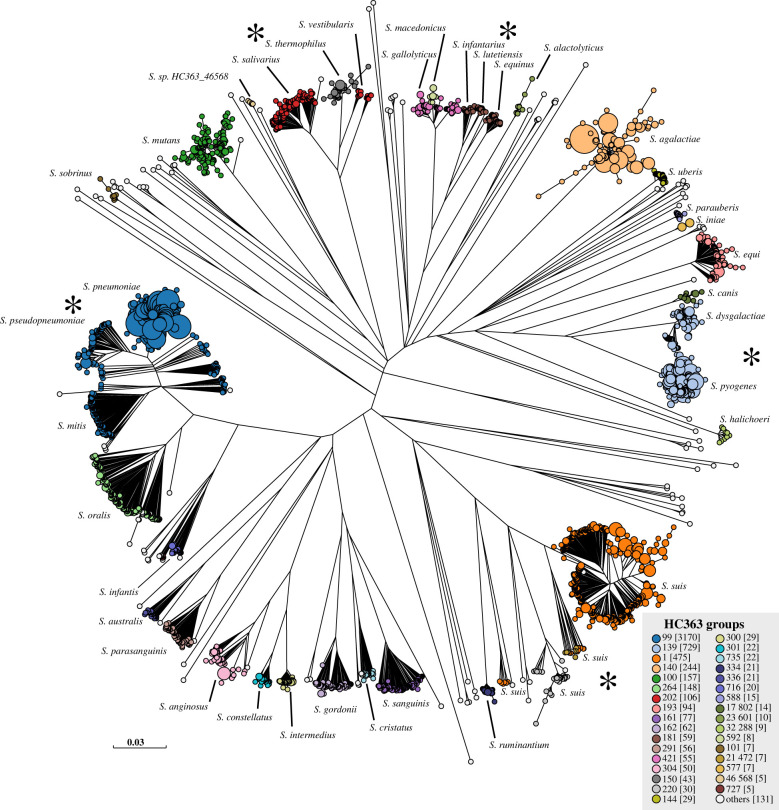
Comparison of HC363 clusters with taxonomic designations in *Streptococcus*. ML super-tree of 263 080 SNPs among 372 core genes from 5937 representative *Streptococcus* genomes (table 3). Species names are indicated next to the phylogenetic clusters according to the locations of genomes from type strains and public metadata. Nodes were coloured by HC363 clusters, and exceptional assignments are indicated by asterisks next to *S. pneumoniae* and *S. pseudopneumoniae*, which were both HC363_99; multiple phylogenetic and HierCC clusters within *S. suis*; *S. salivarius* and *S. vestibularis*, which were both HC353_202; *S. lutetiensis* and *S. equinus*, which were both HC363_181; and *S. dysgalactiae* and *S. pyogenes*, which were both HC363_139. An interactive version of the GrapeTree rendition of the SNP tree can be found at https://enterobase.warwick.ac.uk/ms_tree?tree_id=53261 and the presence/absence tree at https://enterobase.warwick.ac.uk/ms_tree?tree_id=53262.

For all five genera summarized above, clustering according to HierCC was largely concordant with the ML super-trees based on core SNPs or presence/absence of accessory genes. Ninety-five percent ANI and taxonomic designations were also largely concordant with the phylogenetic trees, although to a lesser degree. Similar concordances with the trees based on core SNPs (figure 4) and presence/absence of accessory genes (electronic supplementary material, figure S3) were also found for a majority of the named species within *Streptococcus*. One hundred and two HC363 clusters were concordant with 95% ANI clusters, and each of those HierCC clusters was specific for a single species after eliminating *S. milleri, S. periodonticum* and *S. ursoris* (electronic supplementary material, table S5). These results also demonstrated strong agreement between the two methods with classical taxonomy. However, genomes from a large number of other species within *Streptococcus* were not clustered satisfactorily by either method.

Earlier publications [15,16,77,78], had demonstrated that *Streptococcus mitis* and *oralis* each represents multiple related sequence clusters rather than two single species, and similar results were obtained with 95% ANI clustering [17]. These observations were confirmed by the current data. Of the 102 concordant HC363 and 95% ANI clusters, 23 clusters from *S. cristatus, S. infantis, S. mitis, S. oralis, S. sanguinis* or *S. suis* each corresponded to only a sub-set of the topological diversity within their species. Furthermore, single HC363 clusters from 18 species or groups of species were subdivided extensively by 95% ANI for a total of 155 discrepancies between the two methods (electronic supplementary material, table S6). These discrepant clusters were within the species *S. suis, S. criceti, S. sanguinis*, *S. oralis, S. hyointestinalis, S. parasanguinis, S. australis, S. infantis* and *S. cristatus.* Ninety-five percent ANI was not able to differentiate *S. pneumoniae* from *S. pseudopneumoniae*, *S. pyogenes* from *S. dysgalactiae*, *S. salivarius* from *S. vestibularis*, or between *S. equinus*, *S. infantarius* and *S. lutentiensis.* Contrariwise, three other 95% ANI clusters each encompassed two HC363 clusters (electronic supplementary material, table S7). HC363 could not distinguish *S. pneumoniae* or *S. pseudopneumoniae* from 57 95% ANI clusters within *S. mitis.* In addition to these problems, eight additional species should not have been given a species name because their type strains belonged to one of these chaotic clusters. We conclude that numerous species have been defined on taxonomic grounds that cannot be correctly identified by either 95% ANI or HierCC, and that multiple discrepancies exist between the two approaches within *Streptococcus*.

### (b) Populations, eBurst groups and Lineages

HierCC supports the identification of populations at multiple hierarchical levels ranging from the species/subspecies down to individual transmission chains. We also expected that intermediate HierCC cluster levels could reliably detect the natural populations defined by legacy MLST. Here we focus on examples of such populations from *Salmonella*, *E. coli/Shigella* and *S. pneumonia*, taxa where they have been examined in greatest detail.

#### (i) Salmonella

eBurstGroups (eBGs) defined by legacy MLST in *Salmonella* generally correspond to HierCC HC900 clusters [32]. In some cases, genetically related groups of eBGs correspond to HC2000 clusters [50]; we refer to those as Lineages. By contrast, none of the HierCC levels corresponded consistently with other, prior intra-species subdivisions of *S. enterica* subsp. *enterica* into lineages [79], clades [80–83] or branches [84]. This failure may reflect a high frequency of extensive homologous recombination at these deeper branches, which can obscure topological relationships [51,84], whereas HC900 (and HC2000 clusters) diverged more recently, and have not yet undergone extensive recombination [51].

Traditional subgrouping nomenclature of *Salmonella* below the subspecies level predates DNA sequencing and is predominantly based on serovar designations. Serovar designations consist of common names that have been assigned to a total of more than 2500 unique antigenic formulae based on epitopes within lipopolysaccharide (O antigen) and two alternately expressed flagellar subunits (H1, H2). These antigenic formulae are written in the form O epitopes: H1 epitopes: H2 epitopes in the revised Kauffmann-White scheme [85], and O epitopes are summarized as O serogroups with distinct numeric designations [86] , e.g. O:4 for the O group of serovar Typhimurium [87]. Detailed metabolic maps for lipopolysaccharide (LPS) synthesis have been elucidated for 47 O serogroups [87,88]. Serogroups from natural isolates can be determined serologically by agglutination reactions with specific antisera or *in silico* from genomic sequences with the programs SeqSero [89] or SISTR [90]. In practice, each of these methods has an error rate of a few percent [27,91].

Several hundred legacy eBGs within subsp. *enterica* were relatively uniform for serovar [27], with multiple exceptions, and many serovars were associated with multiple, apparently unrelated eBGs, likely due to the extensive exchange of genes encoding LPS and flagellar epitopes between eBGs early in their evolutionary history. We proposed that legacy MLST was a better metric for identifying the natural populations than serotyping, and could completely replace this traditional method [27]. Our new data indicate that HC900 clusters are even more reliable than legacy eBGs for recognizing natural populations within *Salmonella*.

**Figure 5.**
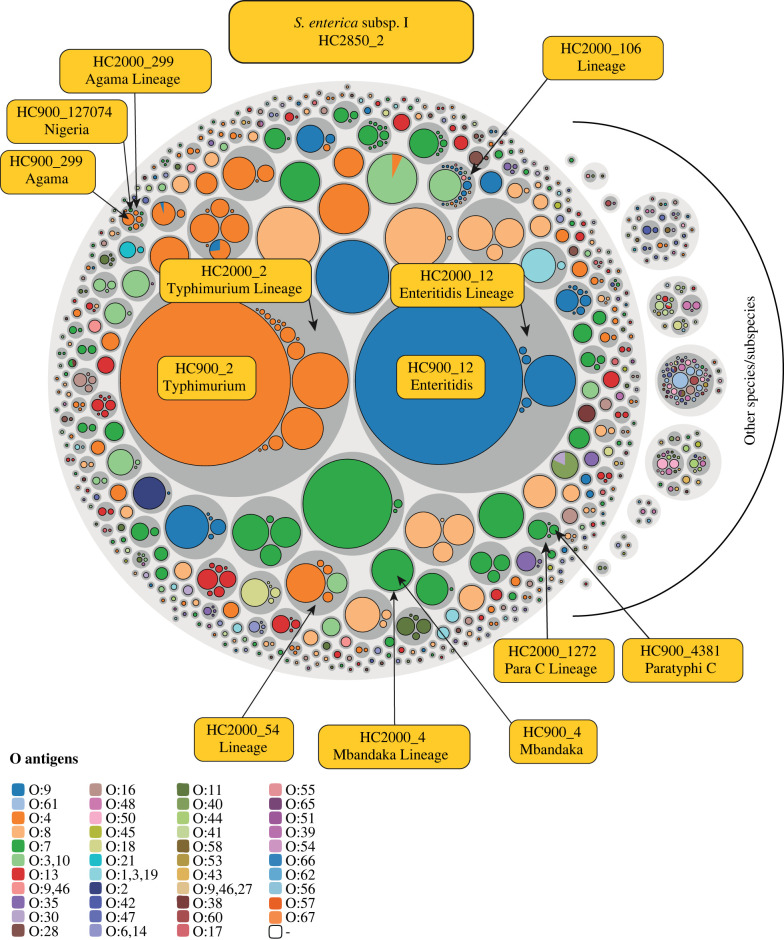
Hierarchical population structure of O serogroups in *Salmonella*. Hierarchical bubble plot of 310 901 *Salmonella* genomes in 790 HC900 clusters for which a consensus O serogroup could be deduced by metadata, or bioinformatic analyses with SeqSero V2 [89] or SISTR 1.1.1 [90]. Taxonomic level (HC level; colours): species/subspecies (HC2850; light grey circles), Lineages (HC2000; dark grey circles) and eBurst groups (HC900; O:group specific colours). Additional information is indicated by yellow text for selected HC2000 and HC900 circles which are specifically mentioned in the text. The diameters of HC900 circles are proportional to the numbers of genomes. An interactive version of this figure can be found at https://observablehq.com/@laurabaxter/salmonella-serovar-piechart from which the representation, raw data and d3 Java code [95] for generating the plot can be downloaded.

Several phylogenetic comparisons indicated that HC900 clusters based on cgMLST are concordant with eBGs in *Salmonella* [32,50]. Here we test this concordance quantitatively over a total of 319 490 genomes (table 4). The concordance between eBG and HC900 clusters was 0.985 according to the Adjusted Mutual Information Score (AMI) (table 4), a metric that is suitable for samples with a heterogeneous size distribution [94], and almost as high with the Adjusted Rand Index (ARI: 0.982), which is less suitable for heterogeneous data. Comparisons of eBG or HC900 with HC2000 clustering yielded slightly lower AMI scores (table 4). We also tested whether HC900 clusters are uniform for serovar. In late 2019, we corrected false serological data in the metadata Serovar field in EnteroBase by manual curation of 790 HC900 clusters that contained at least five entries. Ninety-seven percent (770/790) of those HC900 clusters were uniform (greater than or equal to 95%) for serovar (electronic supplementary material, table S8), as were most HC2000 clusters (437/473 clusters; 92%). The data also indicated that the predominant O groups were uniform over the multiple HC900 clusters within 95% (382/403) of HC2000 clusters within *S. enterica* subsp. *enterica* and 79% (58/70) of HC2000 clusters in other *Salmonella* species and subspecies (figure 5; electronic supplementary material, table S9).

**Table 4.** Quantitative concordance between eBG and HierCC clustering. NOTE: *Salmonella*: Calculations were performed on 319 490 entries in EnteroBase which had been assigned to eBGs, HC900 and HC2000 clusters by December, 2021. The dataset only represented 411 eBGs, 690 HC900 clusters and 312 HC2000 clusters, and consists of a subset of all genomes because new eBGs have not been created in recent years. *Escherichia/Shigella*: HC1100 and HC2000 assignments were from 143 520 genomes which had been assigned to ST Complexes as well as to HC1100 and HC2350 clusters in December, 2021. At that time 186 852 genomes had been assigned to HC1100 and HC2000 clusters, with AMI of 0.59 and ARI of 0.2. *Streptococcus pneumoniae* consisted of GSPC assignments for 18 147 genomes from electronic supplementary material, table S2, and CC assignments for 13 396 genomes from electronic supplementary material, table S1 by Gladstone *et al.* [92] which were imported into User defined fields in EnteroBase as described [28]. ARI and AMI were calculated with the functions adjusted_rand_score() and adjusted_mutual_info_score() in the Python 3 library sklearn.metrics v. 1.0.1 [93].

*Salmonella*			
Test statistic	eBG-HC900	eBG-HC2000	HC900-HC2000
Adjusted Mutual Information Score (AMI)	0.985	0.948	0.942
Adjusted Rand Index (ARI)	0.982	0.856	0.866
*Escherichia/Shigella*			
Test statistic	ST Cplx-HC1100	ST Cplx-HC2000	HC1100-HC2000
Adjusted Mutual Information Score (AMI)	0.940	0.688	0.692
Adjusted Rand Index (ARI)	0.871	0.299	0.337
*Streptococcus pneumoniae*			
Test statistic	CC-HC100	GSPC-HC100	GSPC-HC160
Adjusted Mutual Information Score (AMI)	0.959	0.956	0.980
Adjusted Rand Index (ARI)	0.987	0.906	0.949

HC2000 Lineages with uniform O group included the Para C Lineage (HC2000_1272; serovars Paratyphi C, Typhisuis, Choleraesuis and Lomita [51]) which emerged about 3500 years ago (electronic supplementary information, Supplemental Text) [51,84]. The Para C Lineage is uniformly serogroup O:7, which is not particularly surprising because all its serovars have identical antigenic formulaes, and are only distinguished by biotype. The Enteritidis Lineage (HC2000_12) consists of serovars Enteritidis (1,9,12:g,m:-), Gallinarum and its variant Pullorum (1,9,12:-:-), and Dublin (1,9,12:g,p:-:-) [50] (figure 5), and is uniform for O:9. The Typhimurium Lineage (HC2000_2) includes serovars Typhimurium, Heidelberg, Reading, Saintpaul, Haifa, Stanleyville and others [50]. This Lineage is also uniform for O group because all these serovars are O:4. The Mbandaka Lineage (HC2000_4) includes serovars Mbandaka (6,7,14: z10:e,n,z15) and Lubbock (6,7:g,m,s:e,n,z15), and is uniformly O:7. However, three Lineages were exceptional, and did contain more than one O serogroup. HC2000_299 is a mixture of O:4 (HC900_299; serovar Agama [28]) and O:7 (HC900_127074; serovar Nigeria). HC900 clusters within HC2000_54 are O:4 (HC900_54: Bredeney, Schwarzengrund; HC24937: Kimuenza) or O:9,46 (HC900_57: Give). Similarly, HC900 clusters within HC2000_106 are O:3,10; O:9; O:9,46; O:4: or O:8. These observations confirm and extend the prior conclusions about concordance between natural populations and serogroup within *Salmonella*.

#### (ii) Escherichia coli

Patterns of multilocus isoenzyme electrophoresis were used in the 1980s to provide an overview of the genetic diversity of *E. coli* (see overview by Chaudhuri and Henderson [96]). Those analyses yielded a representative collection of 72 isolates [97], the EcoR collection, whose deep phylogenetic branches were designated haplogroups A, B1, B2, C, D and E [98]. Several haplogroups have since been added [99,100]. The presence or absence of several accessory genes can be used for the assignment of genomes to haplogroups with the Clermont scheme [101,102], and the haplogroup can also be predicted *in silico* from genomic assemblies with ClermontTyping [103] or EZClermont [104], both of which are implemented within EnteroBase. However, the Clermont scheme ignores *Shigella*, which consists of *E. coli* clades despite its differing genus designation [26,47,48], and makes multiple discrepant assignments according to phylogenetic trees [28]. The Clermont scheme also does not properly handle the entire diversity of species and environmental clades/subspecies within the genus *Escherichia* [28,63,64,66].

EnteroBase HierCC automatically assigns genomes within the *Escherichia/Shigella* database to the cgMLST equivalents of ST Complexes (HC1100 clusters) and Lineages (HC2000 clusters) (table 1). It also perpetuates the ST Complexes that were initially defined for legacy MLST by Wirth *et al.* [26]. However, the numbers and composition of legacy ST Complexes have not been updated since 2009 because additional sequencing data defined intermediate, recombinant genotypes that were equidistant to multiple ST Complexes, and threatened to merge existing ST Complexes. By contrast, HC1100 clusters do not merge: intermediate genotypes are rare because cgMLST involves 2512 loci while legacy MLST was based on only seven. Secondly, new genotypes which are similar to and equidistant to multiple clusters do not trigger merging because HierCC arbitrarily assigns them to the oldest of the existing alternatives [32]. Finally, unlike ST Complex designations in legacy MLST, which have not been actively updated in *Escherichia*, HierCC clusters are automatically created as necessary for new genotypes.

**Figure 6.**
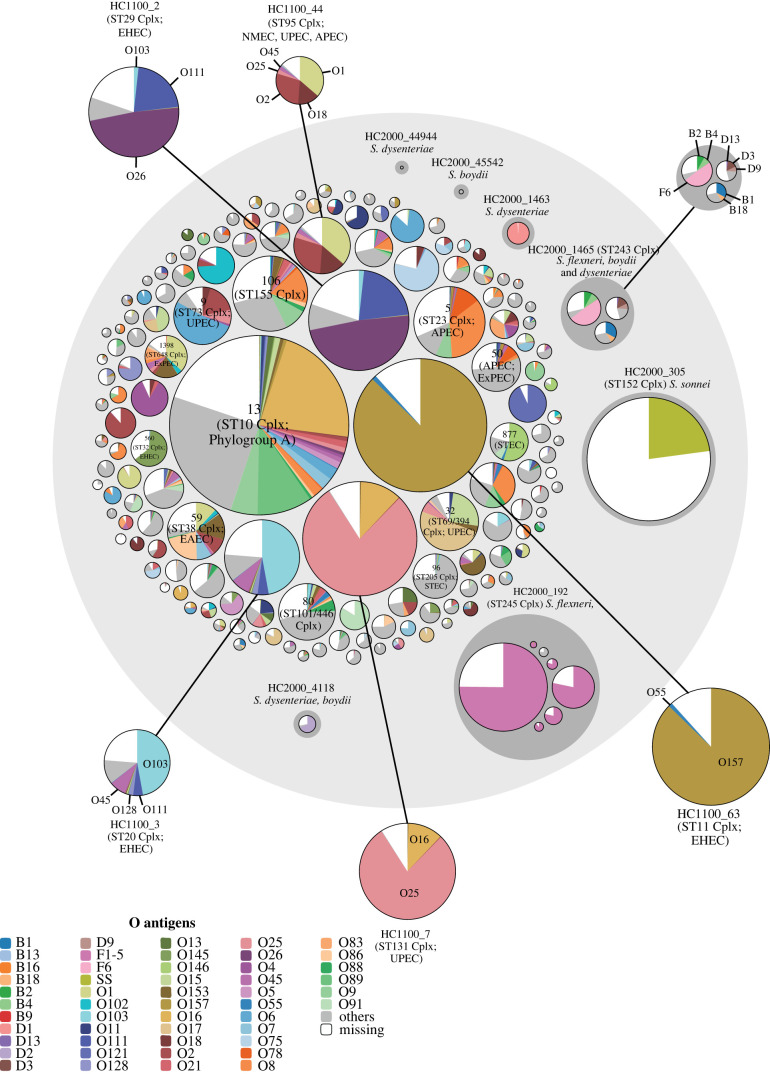
Hierarchical population structure of O serogroups in *Escherichia coli/Shigella*. Hierarchical bubble plot for 167 312 genomes of *Escherichia coli* or *Shigella* in HC2350_1 (large light grey circle) that were available in EnteroBase in April, 2021. Seven HC2000 Lineages encompassing 15 HC1100/ST Complexes of *Shigella* are shown at the right*.* HC2350_1 also includes 15 other *E. coli* HC2000 Lineages that each contains at least 50 *E. coli* genomes and encompass 144 other HC1100 clusters. The remainder of the figure shows those HC1100 clusters and not the corresponding HC2000 clusters. Numbers of genomes assigned to individual O serogroups (legend) are indicated by pie chart wedges within the HC1100 circles. Selected HC1100 clusters are also depicted with indications of phenotype and nomenclature at a larger scale outside the main circle, connected to the original circles by lines. An interactive version of this figure can be found at https://observablehq.com/@laurabaxter/escherichia-serovar-piechart, from which the representation, raw data and d3 Java code [95] for generating the plot can be downloaded.

Frequent recombination in *E. coli* results in poor bootstrap support for the deep branches in phylogenetic trees of concatenated genes [26], and we were unable to identify a unique HierCC level which was largely concordant with haplogroups according to the Clermont scheme. However, similar to *Salmonella,* HC1100 clusters identify clear population groups, which are highly concordant with legacy ST Complexes **(**AMI = 0.94) (table 4). Unlike *Salmonella*, HC2000 clusters did not in general mark recognizable additional population structure beyond that which was provided by ST Complexes, and HC2000 clusters are only moderately concordant with either legacy ST Complexes (AMI = 0.69) or their cgMLST equivalent, HC1100 clusters (AMI = 0.69) (table 4). Multiple *Shigella* species are exceptions to this rule and their legacy ST Complexes [26] equate to cgMLST HC2000 clusters rather than HC1100 clusters according to phylogenetic trees. HC2000_305 replaces ST152 Cplx, and encompasses all *S. sonnei* (figure 6). HC2000_192 replaces ST245 Cplx, and contains many *S. flexneri*. However, *S. flexneri* O group F6 is in an HC1100 cluster within HC2000_1465 (ST243 Cplx), as are *S. boydii* B2 and B4. HC2000_1465 also includes a second HC1100 cluster with *S. boydii* O groups B1 and B18, and a third with *S. dysenteriae* D3, D9 and D13 (figure 6). HC2000_4118 replaces the combination of ST250 Complex and ST149 Complex, which have merged, and contains both *S. dysenteriae* as well as *S. boydii*.

Unlike *Salmonella*, O serogroups are remarkably variable within HC1100 clusters of *E. coli* (figure 6). HC1100_63 (ST11 Complex) combines classical EHEC strains of serovar O157:H7 as well as O55:H7 [105], and other, atypical ancestral EPEC isolates of serovar O55:H7 [106]. Multiple other HC1100 clusters that contain EHEC strains also encompass multiple O groups, including HC1100_2 (ST29 Cplx; O26, O103, O111) [107] and HC1100_3 (ST20 Cplx; O45, O103, O111, O128). Similarly, multiple HC1100 clusters of *E. coli* that cause extra-intestinal diseases also contain a variety of O groups, including HC1100_7 (ST131 Cplx; O16, O25) [23,108,109] and HC1100_44 (K1-encapsulated bacteria of the ST95 Cplx; O1, O2, O18, O25, O45) [26,110,111]. Indeed, the primary impression from figure 6 is that O group variation within an HC1100 cluster is nearly universal throughout *E. coli*, confirming prior conclusions about the high frequency of homologous recombination in this species [26,99].

#### (iii) Other genera

Correlations between intra-specific HierCC clusters and population groupings at multiple levels, including ST Complexes and ribotypes have been described in *Clostridioides difficile* [112]. It remains to be tested whether HierCC can identify populations within *Yersinia* because bacterial populations in *Y. pseudotuberculosis* are largely obscured by recombination [113]. Similarly, we are not aware of extensive efforts to identify bacterial populations which could be compared with HierCC within *Vibrio*. However, recent work has described the assignment of large numbers of genomes of *Streptococcus pneumoniae* to legacy Clonal Complexes as well as GSPC (Global Pneumococcal Sequence Clusters) [92], initially with PopPunk [114] and more recently with Mandrake [115]. Most of those genomes have been assembled within EnteroBase, and assigned to HierCC clusters. We therefore compared the HierCC and GSPC assignments for 18 147 genomes, and also compared them to Clonal Complexes (CCs) based on legacy MLST (13 396 genomes). HC160 clusters yielded the highest AMI and ARI scores with GSPC clusters while CC was most concordant with HC100 clusters (table 4). These conclusions were also supported by visual comparisons of the colour-coding of nodes within a Neighbor-Joining tree based on cgMLST distances according to the various clustering criteria (electronic supplementary material, figure S4). Thus, HC100 clusters seem to be concordant with Clonal Complexes within *S. pneumonia* and Mandrake/PopPunk clustering equates roughly to HC160 clusters.

## 3. Discussion

In 2013, we anticipated that EnteroBase might eventually contain as many as 10 000 genomes of *Salmonella* and *Escherichia*. By the end of 2021 it hosted greater than 600 000 assembled genomes of *Salmonella*, *Escherichia/Shigella*, *Streptococcus*, *Vibrio*, *Clostridioides* and *Yersinia*, and has become one of the primary global sources of their genomic data. An analyses of their genomic properties is facilitated by software tools (table 2) that support indexing of genetic diversity, searching for genomes with specific metadata or genetic relations and investigating stable population structures at multiple levels. Here we focused on the automated identification and designation of species/subspecies and populations on the basis of HierCC of core genome MLST genotypes. The data demonstrate that HierCC can completely replace classical taxonomic methods for genomes of *Salmonella*, *Escherichia/Shigella*, *Vibrio*, *Clostridioides* and *Yersinia*, with only few minor exceptions. HierCC is also a complete replacement for legacy MLST regarding the assignments to populations within *Salmonella*, *Escherichia/Shigella* and *Streptococcus pneumoniae*.

### (a) Taxonomic assignments

The paired ML super-trees based on core SNPs and presence/absence of accessory genes from each genus were generally concordant in their clustering patterns, indicating that those genera contain well-defined and reproducible species/subspecies independent of the genetic criteria. Those phylogenetic clusterings were then used to resolve the most appropriate designations for genomes which were assigned contradictory designations by their taxonomic metadata *versus* clusters based on 95% ANI or HierCC. The frequency of agreement with current taxonomy was comparable for HierCC and 95% ANI at the species level within *Salmonella*, *Escherichia/Shigella*, *Vibrio*, *Clostridioides* and *Yersinia* but 95% ANI was generally unable to resolve subspecies whereas HierCC did not distinguish between subspecies and species clusters. Classical taxonomical designations exhibited multiple, glaring problems in each genus and we replaced those problematical designations in EnteroBase with labels based on the automated, HierCC-based species/subspecies taxonomy. We also defined novel species/subspecies, and labelled them with HierCC-based designations.

Why were there so many obvious discrepancies between the different approaches? One obvious reason is that we have discarded the medical tradition of retaining discrete species names for pathogens that cause particular diseases, and recommend designating *Y. pestis* as a subspecies of *Y. pseudotuberculosis.* Other discrepancies may reflect technical errors in manipulating data or uploading information to public databases, or simple strain mix-ups [46,50]. Possibly the discrepancies were particularly obvious because our analyses were performed at an unprecedented scale over multiple species. Many insights can be attributed to the facile ability to investigate large databases within a graphic framework that is provided by EnteroBase, and to our optimization of HierCC levels for each genus. However, we failed in a similar attempt to optimize ANI levels for recognizing both species and subspecies.

Our taxonomic changes in *Yersinia* are likely to be highly controversial*.* According to both ML trees and HierCC, the current designation ‘*Y. enterocolitica*’ encompasses four, previously unnamed species/subspecies. These taxa are not distinguished by 95% ANI and had not previously been assigned distinct names. We maintain continuity with the traditional designation of *Y. enterocolitica*, and simply add HierCC affiliations to the species name, e.g. *Y. enterocolitica* HC1490_2. Alternatively, these clusters of strains could be considered to represent subspecies and their name modified slightly, e.g. *Y. enterocolitica* subspecies HC1490_2, similar to our downgrading of the named species *Y. pestis*, *Y. similis* [74] and *Y. wautersii* [73] on phylogenetic grounds to subspecies of *Y. pseudotuberculosis* (electronic supplementary material, figure S1)*.* Finally, we defined eight new species/subspecies within *Yersinia* without submitting traditional evidence for simple phenotypic differences, and named them after their HC cluster, e.g. *Yersinia HC1490_4399*. We also followed comparable strategies for the other genera analysed here.

In *Salmonella*, HierCC and 95% ANI identified a new species, *Salmonella HC2850_215890*, and a new subspecies, *S. enterica* subsp. HC2850_222931 (figure 1). Five new species were identified within *C. difficile* by HierCC as well as ANI, and HierCC identified two additional new subspecies. In *Escherichia* we provide HierCC designations for multiple clusters of bacteria from environmental sources near Hong Kong that were previously unnamed.

The ML trees presented here (electronic supplementary material, figure S2) support prior definitions of high order clades containing multiple species in *Vibrio* on the basis of MLSA [76]. We also observed a general concordance between ANI and HierCC for 109 named *Vibrio* species. However, 43 HierCC clusters of species rank had not previously been properly classified with species designations, and 17 other species names were superfluous. These changes are fully described in the electronic supplementary material (text), allowing closer scrutiny by the *Vibrio* taxonomic community for consistency with other properties.

HierCC was so effective in clarifying the taxonomies of these five species that we had hoped that it could also facilitate the taxonomic classification of *Streptococcus.* One hundred and two HC363 clusters were indeed concordant with 95% ANI and taxonomic designations, with only minor exceptions. However, extensive discrepancies existed between 95% ANI and HierCC for numerous other clusters, and both approaches showed multiple discrepancies with classical taxonomic designations. The existence of multiple 95% ANI clusters within *S. mitis* and *S. oralis* and other species of *Streptococcus* has previously been commented on by Kilian and his colleagues [15,16,77,78]. Our observations extend these taxonomic problems to multiple additional species in which HC363 does not distinguish between pairs of named species (figure 4). Neither 95% ANI nor cgMLST HierCC seems to provide a suitable general strategy for elucidating the taxonomy of all of *Streptococcus*, and we failed to find a general solution to this problem.

### (b) HierCC versus classical taxonomy

Classical microbiological species taxonomy involves identifying a type strain whose phenotype can be distinguished from all other type strains, identifying additional isolates with similar phenotypes, demonstrating distinct clustering from other known species in phylogenetic trees based on DNA or amino acid sequences, and publishing a report in one of a very limited number of acceptable journals. Such species definitions are then considered tentative until an international committee has approved them. Other forms of identifying species that include sole reliance on DNA sequence differences are not acceptable [7,8]. The metagenomics community and scientists working with uncultivated organisms from the environment have largely liberated themselves from such regulations, and tend to use operational taxonomic units (OTUs) as taxonomic entities. However, the taxonomic species structure of many microbes from environmental sources remains fuzzy, or does not clearly correspond to classical taxonomy (e.g. *Prochlorococcus* [116] or *Synechococcus* [117]). Furthermore, we are not aware of any other method that is able to assign 1000s of genomes per day to existing taxa and also reliably identify new taxa automatically as they appear. HierCC performs this task with bravura for the genera in table 1, with the notable exception of *Streptococcus*.

We have taken the liberty of dropping all attempts to reconcile HierCC species/subspecies clusters with classical prescriptions for how to define a species. Instead we have adopted the practice of using HierCC cluster designations within EnteroBase for the nomenclatures for species/subspecies groupings that had not yet been identified by others and eliminated from EnteroBase multiple species designations of type strains in *Vibrio* and *Streptococcus* which did not match the ML tree topologies and HierCC clusters. These actions provide a uniform base for the future additions of additional species and ensured that future genomes can be correctly assigned to uniform clusters of related strains. Scientists wishing to use these schemes to identify the species of their bacterial isolates can upload their sequenced genomes to EnteroBase. The HierCC assignments will be available within hours. We also welcome additional curators of these databases who are willing to test and improve the current taxonomic assignments we have implemented. But we reject the concept of designating our assignments as tentative until they are confirmed in several years by an international committee.

### (c) HierCC and populations

The first bacterial taxonomic designations were assigned over 100 years ago. Bacterial population genetics is much younger and has many fewer practitioners. Initial population genetic analyses in the early 1980s subdivided multiple bacterial species into intra-specific lineages based on multilocus enzyme electrophoresis [97]. This methodology was replaced in the late 1990s by legacy MLST based on several housekeeping genes [21], which is currently being replaced by cgMLST based on all genes in the soft core genome [22,29]. EnteroBase calculates genotypes for both types of MLST, as well as for rMLST [28]. Legacy STs differ from cgSTs due to different levels of resolution. However, the boundaries of ST Complexes/eBGs are highly concordant with HierCC clusters, with AMI indices of 0.985 for eBGs versus HC900 *in Salmonella* and 0.94 for ST Complexes versus HC1100 clusters in *Escherichia/Shigella* (table 4). We recommended previously that serovars should be replaced by eBGs in *S. enterica* [27] and the data presented here show that HC900 clusters are an even better replacement. Our data also indicate that Lineages in *S. enterica* correspond to HC2000 clusters, and that these tend to be highly uniform for O group. The data presented here also indicate that HC1100 groups are a good replacement for detecting populations within *Escherichia* as are HC100 clusters for CCs in *S. pneumoniae* [92] (table 4).

We interpret these consistencies between legacy MLST and cgMLST as reflecting the existence of natural populations. We previously claimed that legacy ST Complexes in *E. coli* were unstable due to frequent recombination [26], and were therefore not surprised at their tendency to merge as additional isolates were sequenced. However, legacy MLST is based on only seven genes. The high resolution of cgMLST and the decision to assign new genotypes that are equidistant from multiple HierCC clusters to the oldest cluster have largely negated these problems, and *E. coli* HC1100 clusters correspond to bacterial groupings that have been independently identified by multiple phenotypic patterns. In the early 1980s, MA began his academic research on bacterial pathogens with *E. coli* that expressed the K1 polysaccharide capsule [110]. He observed unusually uniform patterns of electrophoretic mobility of major outer membrane proteins across multiple isolates, and interpreted those bacteria as representing **‘**clones’. These **‘**clones’ correspond to HC1100 clusters, including HC1100_44 (ST95 Complex) which continues to cause invasive disease in humans and animals around the globe [111]. The 1980s analyses showed that these K1 bacteria were variably O1, O2 or O18, and that the serotype variants differed both in their invasiveness and in the hosts which they infected [118]. As indicated in figure 6, HC1100_44 includes O groups O25 and O45 in addition to O1, O2 and O18. Similarly, HC1100_7 (ST131) represents another major cause of extra-intestinal disease in humans [23,108,109]. EHEC bacteria are notorious for their association with hemolytic uremic syndrome (HUS). Many of them belong to distinctive HC1100 clusters, including O157:H7/O55:H7 (HC1100_63, ST11 Cplx [106,119]) and O26, O103 and O111 EHEC bacteria (HC1100_2, ST29 Cplx [107]) (figure 5).

By contrast to these correlations with epidemiological groupings, we were unable to identify any other HierCC levels that were consistently concordant with other intra-specific phylogenetic subdivisions based on SNP trees, including haplogroups [98] or Clermont typing [101,102] in *E. coli* [28] and clades [80–83], Lineages [79] or branches [84] in *S. enterica.* Instead, we found that a subdivision we refer to as Lineages is marked by some HC2000 clusters in *Salmonella* and *Escherichia/Shigella*.

### (d) Lineages

In *E. coli*, HC2000 Lineages were particularly appropriate for defining the clustering level of seven groups of *Shigella* genomes (figure 6). Lineage designations did not add additional insights into other populations because they were almost entirely encompassed by HC1100 clusters or occasionally even lower level clusters, such as HC400. In *S. enterica* subspecies *enterica*, multiple HC2000 lineages corresponded to genetically related combinations of multiple HC900 clusters, in some cases with distinctive serovar designations. However, all but three of the major Lineages were predominantly uniform in O group. The three exceptional HC2000 Lineages (HC2000_54, HC2000_106, HC2000_299) might be worth exploring in greater detail to reconstruct the recombination that has resulted in their differing LPS epitopes.

HC160 clusters, the Lineage equivalent in *S. pneumonia,* were strongly concordant with PopPunk GSPC clustering. Otherwise, little is yet known about the general properties of Lineages in other genera, except that their properties are likely to vary with species or genus. For example, *S. enterica* HC2000 Lineages were uniform for O group whereas O serogroups were already heterogeneous within HC1100 clusters/ST Complexes within *E. coli* (figure 6).

### (e) Future prospects

EnteroBase was conceived to satisfy a need that MA and ZZ perceived in 2014 [1]. We contend that it is now fit for purpose for investigations of multiple bacterial genera by scientists ranging from beginners through to experts in the areas of microbial epidemiology and population genetics. Its original creators have now all left this project, but EnteroBase is being maintained as a service for the global community by the University of Warwick. Maintenance of its technical functions and databases are thereby assured for the near future. Further functional developments will, however, depend on increased participation and perception of ownership by its users. We perceive a general trend to focus on insular solutions that can satisfy the demands of individual bioinformaticians and regional diagnostic laboratories. Such approaches can yield relatively rapid progress in solving short-term needs. However, a global overview of genomic diversity needs central databases to ensure definitive terminology. Pooling efforts on a central endeavour at the scale represented by EnteroBase would ensure that it continues to function over decades, is representative over all continents, and serves the global community even better. We therefore welcome additional curators and scientific experts as well as bioinformatics collaborations to help improve EnteroBase even more.

## 4. Methods (see supplemental text)

## Data Availability

cgMLSA is available for downloading at https://github.com/zheminzhou/cgMLSA. All trees in figures and genomic data are freely available within EnteroBase for interactive examination. Other code is described in electronic supplementary material, text. Datasets used for the analyses presented here have been deposited for permanent public access at http://wrap.warwick.ac.uk/162247/. The data are provided in electronic supplementary material [120].

## References

[1] Achtman M, Zhou Z. 2014 Distinct genealogies for plasmids and chromosome. PLoS Genet. **10**, e1004874. (10.1371/journal.pgen.1004874) 25521852 PMC4270482

[2] Kauffmann F. 1961 Die Bakteriologie der *Salmonella*-Species. Copenhagen, Denmark: Munksgaard.

[3] Morelli G et al. 2010 *Yersinia pestis* genome sequencing identifies patterns of global phylogenetic diversity. Nature Genet. **42**, 1140-1143. (10.1038/ng.705) 21037571 PMC2999892

[4] Achtman M, Zurth K, Morelli G, Torrea G, Guiyoule A, Carniel E. 1999 *Yersinia pestis*, the cause of plague, is a recently emerged clone of *Yersinia pseudotuberculosis*. Proc. Natl Acad. Sci. USA **96**, 14 043-14 048. (10.1073/pnas.96.24.14043) 10570195 PMC24187

[5] Pulford CV et al. 2021 Stepwise evolution of *Salmonella* Typhimurium ST313 causing bloodstream infection in Africa. Nat. Microbiol. **6**, 327-338. (10.1038/s41564-020-00836-1) 33349664 PMC8018540

[6] Wayne LG et al. 1987 Report of the ad hoc committee on reconciliation of approaches to bacterial systematics. Int. J. Syst. Bacteriol. **37**, 463-464. (10.1099/00207713-37-4-463)

[7] Murray AE et al. 2020 Roadmap for naming uncultivated Archaea and Bacteria. Nat. Microbiol. **5**, 987-994. (10.1038/s41564-020-0733-x) 32514073 PMC7381421

[8] Sutcliffe IC, Dijkshoorn L, Whitman WB and Executive Board. 2020 Minutes of the International Committee on Systematics of Prokaryotes online discussion on the proposed use of gene sequences as type for naming of prokaryotes, and outcome of vote. Int. J. Syst. Evol. Microbiol. **70**, 4416-4417. (10.1099/ijsem.0.004303) 32628106 PMC7657488

[9] Konstantinidis KT, Tiedje JM. 2005 Genomic insights that advance the species definition for prokaryotes. Proc. Natl Acad. Sci. USA **102**, 2567-2572. (10.1073/pnas.0409727102) 15701695 PMC549018

[10] Jain C, Rodriguez R, Phillippy AM, Konstantinidis KT, Aluru S. 2018 High throughput ANI analysis of 90 K prokaryotic genomes reveals clear species boundaries. Nat. Commun. **9**, 5114. (10.1038/s41467-018-07641-9) 30504855 PMC6269478

[11] Auch AF, von Jan M, Klenk HP, Göker M. 2010 Digital DNA-DNA hybridization for microbial species delineation by means of genome-to-genome sequence comparison. Stand. Genom. Sci. **2**, 117-134. (10.4056/sigs.531120) PMC3035253 21304684

[12] Garrido-Sanz D, Meier-Kolthoff JP, Goker M, Martin M, Rivilla R, Redondo-Nieto M. 2016 Genomic and genetic diversity within the *Pseudomonas fluorescens* complex. PLoS ONE **11**, e0150183. (10.1371/journal.pone.0150183) 26915094 PMC4767706

[13] Richter M, Rosselló-Mora R. 2009 Shifting the genomic gold standard for the prokaryotic species definition. Proc. Natl Acad. Sci. USA **106**, 19 126-19 131. (10.1073/pnas.0906412106) PMC2776425 19855009

[14] Maderankova D, Jugas R, Sedlar K, Vitek M, Skutkova H. 2019 Rapid bacterial species delineation based on parameters derived from genome numerical representations. Comput. Struct. Biotechnol. J. **17**, 118-126. (10.1016/j.csbj.2018.12.006) 30728919 PMC6352304

[15] Jensen A, Scholz CF, Kilian M. 2016 Re-evaluation of the taxonomy of the Mitis group of the genus *Streptococcus* based on whole genome phylogenetic analyses, and proposed reclassification of *Streptococcus dentisani* as *Streptococcus oralis* subsp. *dentisani* comb. nov., *Streptococcus tigurinus* as *Streptococcus oralis* subsp. *tigurinus* comb. nov., and *Streptococcus oligofermentans* as a later synonym of *Streptococcus cristatus*. Int. J. Syst. Evol. Microbiol. **66**, 4803-4820. (10.1099/ijsem.0.001433) 27534397

[16] Kilian M, Poulsen K, Blomqvist T, Havarstein LS, Bek-Thomsen M, Tettelin H, Sorensen UB. 2008 Evolution of *Streptococcus pneumoniae* and its close commensal relatives. PLoS ONE **3**, e2683. (10.1371/journal.pone.0002683) 18628950 PMC2444020

[17] Zhou Z, Charlesworth J, Achtman M. 2020 Accurate reconstruction of bacterial pan- and core genomes with PEPPAN. Genome Res. **30**, 1667-1679. (10.1011/gr.260828.120) 33055096 PMC7605250

[18] Gomila M, Pena A, Mulet M, Lalucat J, Garcia-Valdes E. 2015 Phylogenomics and systematics in *Pseudomonas*. Front. Microbiol. **6**, 214. (10.3389/fmicb.2015.00214) 26074881 PMC4447124

[19] Beaz-Hidalgo R, Hossain MJ, Liles MR, Figueras MJ. 2015 Strategies to avoid wrongly labelled genomes using as example the detected wrong taxonomic affiliation for *Aeromonas* genomes in the GenBank database. PLoS ONE **10**, e0115813. (10.1371/journal.pone.0115813) 25607802 PMC4301921

[20] Achtman M, Wagner M. 2008 Microbial diversity and the genetic nature of microbial species. Nat. Rev. Microbiol. **6**, 431-440. (10.1038/nrmicro1872) 18461076

[21] Maiden MCJ et al. 1998 Multilocus sequence typing: a portable approach to the identification of clones within populations of pathogenic microorganisms. Proc. Natl Acad. Sci. USA **95**, 3140-3145. (10.1073/pnas.95.6.3140) 9501229 PMC19708

[22] Jolley KA, Bray JE, Maiden MC. 2018 Open-access bacterial population genomics: BIGSdb software, the PubMLST.org website and their applications. Wellcome Open Res. **3**, 124. (10.12688/wellcomeopenres.14826.1) 30345391 PMC6192448

[23] Stoesser N et al. 2016 Evolutionary history of the global emergence of the *Escherichia coli* epidemic clone ST131. MBio **7**, e02162. (10.1128/mBio.02162-15) 27006459 PMC4807372

[24] Kingsley RA et al. 2009 Epidemic multiple drug resistant *Salmonella* Typhimurium causing invasive disease in sub-Saharan Africa have a distinct genotype. Genome Res. **19**, 2279-2287. (10.1101/gr.091017.109) 19901036 PMC2792184

[25] Feil EJ, Li BC, Aanensen DM, Hanage WP, Spratt BG. 2004 eBURST: inferring patterns of evolutionary descent among clusters of related bacterial genotypes from Multilocus Sequence Typing data. J. Bacteriol. **186**, 1518-1530. (10.1128/JB.186.5.1518-1530.2004) 14973027 PMC344416

[26] Wirth T et al. 2006 Sex and virulence in *Escherichia coli*: an evolutionary perspective. Mol. Microbiol. **60**, 1136-1151. (10.1111/j.1365-2958.2006.05172.x) 16689791 PMC1557465

[27] Achtman M et al. 2012 Multilocus sequence typing as a replacement for serotyping in *Salmonella enterica*. PLoS Pathog. **8**, e1002776. (10.1371/journal.ppat.1002776) 22737074 PMC3380943

[28] Zhou Z, Alikhan NF, Mohamed K, Fan Y, Achtman M. 2020 The EnteroBase user's guide, with case studies on *Salmonella* transmissions, *Yersinia pestis* phylogeny, and *Escherichia* core genomic diversity. Genome Res. **30**, 138-152. (10.1101/gr.251678.119) 31809257 PMC6961584

[29] Maiden MC, van Rensburg MJ, Bray JE, Earle SG, Ford SA, Jolley KA, McCarthy ND. 2013 MLST revisited: the gene-by-gene approach to bacterial genomics. Nat. Rev. Microbiol. **11**, 728-736. (10.1038/nrmicro3093) 23979428 PMC3980634

[30] Alikhan N-F, Zhou Z, Sergeant MJ, Achtman M. 2018 A genomic overview of the population structure of *Salmonella*. PLoS Genet. **14**, e1007261. (10.1371/journal.pgen.1007261) 29621240 PMC5886390

[31] Moura A et al. 2016 Whole genome-based population biology and epidemiological surveillance of *Listeria monocytogenes*. Nat. Microbiol. **2**, 16185. (10.1038/nmicrobiol.2016.185) 27723724 PMC8903085

[32] Zhou Z, Charlesworth J, Achtman M. 2021 HierCC: a multi-level clustering scheme for population assignments based on core genome MLST. Bioinformatics **37**, 3645-3646. (10.1093/bioinformatics/btab234) PMC8545296 33823553

[33] Griffiths D et al. 2010 Multilocus sequence typing of *Clostridium difficile*. J. Clin. Microbiol. **48**, 770-778. (10.1128/JCM.01796-09) 20042623 PMC2832416

[34] Zhou Z, Alikhan N-F, Sergeant MJ, Luhmann N, Vaz C, Francisco AP, Carrico JA, Achtman M. 2018 GrapeTree: visualization of core genomic relationships among 100,000 bacterial pathogens. Genome Res. **28**, 1395-1404. (10.1101/gr.232397.117) 30049790 PMC6120633

[35] Achtman M, Zhou Z. 2020 Metagenomics of the modern and historical human oral microbiome with phylogenetic studies on *Streptococcus mutans* and *Streptococcus sobrinus*. Phil. Trans. R. Soc. B **375**, 20190573. (10.1098/rstb.2019.0573) 33012228 PMC7702799

[36] Zhou Z, Luhmann N, Alikhan N-F, Quince C, Achtman M. 2018 Accurate reconstruction of microbial strains from metagenomic sequencing using representative reference genomes. In RECOMB 2018, pp. 225-240. Cham, Switzerland: Springer.

[37] Luhmann N, Holley G, Achtman M. 2021 BlastFrost: fast querying of 100,000s of bacterial genomes in Bifrost graphs. Genome Biol. **22**, 30. (10.1186/s13059-020-02237-3) 33430919 PMC7798312

[38] Jones G et al. 2019 Outbreak of *Salmonella enterica* serotype Poona in infants linked to persistent *Salmonella* contamination in an infant formula manufacturing facility, France, August 2018 to February 2019. Euro Surveill. **24**, 13. (10.2807/1560-7917.ES.2019.24.13.1900161) PMC6446512 30940315

[39] Jones G et al. 2019 Outbreak of *Shiga* toxin-producing *Escherichia coli* (STEC) O26 paediatric haemolytic uraemic syndrome (HUS) cases associated with the consumption of soft raw cow's milk cheeses, France, March to May 2019. Euro Surveill. **24**, 1900305. (10.2807/1560-7917.ES.2019.24.22.1900305) PMC6549459 31164190

[40] Van den Bossche A, Ceyssens PJ, Denayer S, Hammami N, van den Beld M, Dallman TJ, Mattheus W. 2021 Outbreak of Central American born *Shigella sonnei* in two youth camps in Belgium in the summer of 2019. Eur. J. Clin. Microbiol. Infect. Dis. **40**, 1573-1577. (10.1007/s10096-021-04164-y) 33569724 PMC8205880

[41] European Centre for Disease Prevention and Control, EFSA. 2020 Multi-country outbreak of *Salmonella* Typhimurium and *S.* Anatum infections linked to Brazil nuts - 21 October 2020. EFSA Supporting Publications **17**, 1944E. (10.2903/sp.efsa.2020.EN-1944)

[42] European Food Safety Authority European Centre for Disease Prevention and Control. 2022 Multi-country outbreak of monophasic *Salmonella typhimurium* sequence type 34 linked to chocolate products: first update, 18 May 2022. *EFSA Supporting Publications* 19 (6): 7352E.

[43] Hawkey J et al. 2021 Global population structure and genotyping framework for genomic surveillance of the major dysentery pathogen, *Shigella sonnei*. Nat. Commun. **12**, 2684. (10.1038/s41467-021-22700-4) 33976138 PMC8113504

[44] Park SE et al. 2018 The phylogeography and incidence of multi-drug resistant typhoid fever in sub-Saharan Africa. Nat. Commun. **9**, 5094. (10.1038/s41467-018-07370-z) 30504848 PMC6269545

[45] Weill FX et al. 2017 Genomic history of the seventh pandemic of cholera in Africa. Science **358**, 785-789. (10.1126/science.aad5901) 29123067

[46] Achtman M, van den Broeck F, Cooper KK, Lemey P, Parker CT, Zhou Z, and the ATCC14028s Study Group. 2021 Genomic population structure associated with repeated escape of *Salmonella enterica* ATCC14028s from the laboratory into nature. PLoS Genet. **17**, e1009820. (10.1371/journal.pgen.1009820) 34570761 PMC8496778

[47] Yassine I et al. 2022 Population structure analysis and laboratory monitoring of *Shigella* by core-genome multilocus sequence typing. Nat. Commun. **13**, 551. (10.1038/s41467-022-28121-1) 35087053 PMC8795385

[48] Pupo GM, Lan R, Reeves PR. 2000 Multiple independent origins of *Shigella* clones of *Escherichia coli* and convergent evolution of many of their characteristics. Proc. Natl Acad. Sci. USA **97**, 10 567-10 572. (10.1073/pnas.180094797) PMC27065 10954745

[49] Zhang X, Payne M, Nguyen T, Kaur S, Lan R. 2021 Cluster-specific gene markers enhance *Shigella* and enteroinvasive *Escherichia coli in silico* serotyping. Microb. Genom. **7**, 000704. (10.1099/mgen.0.000704) PMC8767346 34889728

[50] Achtman M et al. 2020 Genomic diversity of *Salmonella enterica*—the UoWUCC 10K genomes project. Wellcome Open Res. **5**, 223. (10.12688/wellcomeopenres.16291.2) 33614977 PMC7869069

[51] Zhou Z et al. 2018 Pan-genome analysis of ancient and modern *Salmonella enterica* demonstrates genomic stability of the invasive Para C lineage for millennia. Curr. Biol. **28**, 2420-2428. (10.1016/j.cub.2018.05.058) 30033331 PMC6089836

[52] Zaharias P, Warnow T. 2022 Recent progress on methods for estimating and updating large phylogenies. Phil. Trans. R. Soc. B **377**, 2021100258. (10.20944/preprints202110.0258.v1) PMC9393559 35989607

[53] Vachaspati P, Warnow T. 2015 ASTRID: Accurate Species TRees from Internode Distances. BMC Genom. **16**(Suppl. 10), S3. (doi:1471-2164-16-S10-S3) 10.1186/1471-2164-16-S10-S3 PMC4602181 26449326

[54] Sayyari E, Mirarab S. 2016 Fast coalescent-based computation of local branch support from quartet frequencies. Mol. Biol. Evol. **33**, 1654-1668. (10.1093/molbev/msw079) 27189547 PMC4915361

[55] Pearce ME, Langridge GC, Lauer AC, Grant K, Maiden MCJ, Chattaway MA. 2021 An evaluation of the species and subspecies of the genus *Salmonella* with whole genome sequence data: proposal of type strains and epithets for novel *S. enterica* subspecies VII, VIII, IX, X and XI. Genomics **113**, 3152-3162. (10.1016/j.ygeno.2021.07.003) 34242711 PMC8426187

[56] Huys G, Cnockaert M, Janda JM, Swings J. 2003 *Escherichia albertii* sp. nov., a diarrhoeagenic species isolated from stool specimens of Bangladeshi children. Int. J. Syst. Evol. Microbiol. **53**, 807-810. (10.1099/ijs.0.02475-0) 12807204

[57] Hyma KE, Lacher DW, Nelson AM, Bumbaugh AC, Janda JM, Strockbine NA, Young VB, Whittam TS. 2005 Evolutionary genetics of a new pathogenic *Escherichia* species: *Escherichia albertii* and related *Shigella boydii* strains. J. Bacteriol. **187**, 619-628. (10.1128/JB.187.2.619-628.2005) 15629933 PMC543563

[58] Ooka T et al. 2015 Defining the genome features of *Escherichia albertii*, an emerging enteropathogen closely related to *Escherichia coli*. Genome Biol. Evol. **7**, 3170-3179. (10.1093/gbe/evv211) 26537224 PMC4700944

[59] Farmer III JJ, Fanning GR, Davis BR, O'Hara CM, Riddle C, Hickman-Brenner FW, Asbury MA, Lowery III VA, Brenner DJ. 1985 *Escherichia fergusonii* and *Enterobacter taylorae*, two new species of Enterobacteriaceae isolated from clinical specimens. J. Clin. Microbiol. **21**, 77-81. (10.1128/jcm.21.1.77-81.1985) 3968204 PMC271579

[60] Gaastra W, Kusters JG, Van Duijkeren E, Lipman LJ. 2014 Escherichia fergusonii. Vet. Microbiol. **172**, 7-12. (10.1016/j.vetmic.2014.04.016) 24861842

[61] Liu S, Jin D, Lan R, Wang Y, Meng Q, Dai H, Lu S, Hu S, Xu J. 2015 *Escherichia marmotae *sp.* nov*., isolated from faeces of *Marmota himalayana*. Int. J. Syst. Evol. Microbiol. **65**, 2130-2134. (10.1099/ijs.0.000228) 25851592

[62] Liu S et al. 2019 Genomic and molecular characterisation of *Escherichia marmotae* from wild rodents in Qinghai-Tibet plateau as a potential pathogen. Sci. Rep. **9**, 10619. (10.1038/s41598-019-46831-3) 31337784 PMC6650469

[63] van der Putten BCL, Matamoros S, COMBAT Consortium, and Schultsz C. 2021 *Escherichia ruysiae* sp. nov., a novel Gram-stain-negative bacterium, isolated from a faecal sample of an international traveller. Int. J. Syst. Evol. Microbiol. **71**, 004609. (10.1099/ijsem.0.004609) PMC8346766 33406029

[64] Walk ST. 2015 The ‘cryptic’ *Escherichia*. EcoSal Plus **6**, 0002. (10.1128/ecosalplus.ESP-0002-2015) PMC11575852 26435255

[65] Vignaroli C, Di SL, Magi G, Luna GM, Di CA, Pasquaroli S, Facinelli B, Biavasco F. 2015 Adhesion of marine cryptic *Escherichia* isolates to human intestinal epithelial cells. ISME J. **9**, 508-515. (10.1038/ismej.2014.164) 25216085 PMC4303642

[66] Shen Z, Koh XP, Yu Y, Woo CF, Tong Y, Lau SCK. 2019 Draft genome sequences of 16 strains of *Escherichia* cryptic clade II isolated from intertidal sediment in Hong Kong. Microbiol. Resour. Announc. **8**, e00415-19. (10.1128/MRA.00416-19) 31320428 PMC6639607

[67] Luo C, Walk ST, Gordon DM, Feldgarden M, Tiedje JM, Konstantinidis KT. 2011 Genome sequencing of environmental *Escherichia coli* expands understanding of the ecology and speciation of the model bacterial species. Proc. Natl Acad. Sci. USA **108**, 7200-7205. (10.1073/pnas.1015622108) 21482770 PMC3084108

[68] Walk ST, Alm EW, Gordon DM, Ram JL, Toranzos GA, Tiedje JM, Whittam TS. 2009 Cryptic lineages of the genus *Escherichia*. Appl. Environ. Microbiol. **75**, 6534-6544. (10.1128/AEM.01262-09) 19700542 PMC2765150

[69] Walk ST, Alm EW, Calhoun LM, Mladonicky JM, Whittam TS. 2007 Genetic diversity and population structure of *Escherichia coli* isolated from freshwater beaches. Environ. Microbiol. **9**, 2274-2288. (10.1111/j.1462-2920.2007.01341.x) 17686024

[70] Shen ZY, Koh XP, Yu YP, Lau SCK. 2020 Genetic variation and preliminary indications of divergent niche adaptation in cryptic clade II of *Escherichia*. Microorganisms **8**, 1713. (10.3390/microorganisms8111713) PMC7716201 33142902

[71] Knight DR et al. 2021 Major genetic discontinuity and novel toxigenic species in *Clostridioides difficile* taxonomy. Elife **10**, e64325. (10.7554/eLife.64325) 34114561 PMC8241443

[72] Reuter S et al. 2014 Parallel independent evolution of pathogenicity within the genus *Yersinia*. Proc. Natl Acad. Sci. USA **111**, 6768-6773. (10.1073/pnas.1317161111) 24753568 PMC4020045

[73] Savin C et al. 2014 The *Yersinia pseudotuberculosis* complex: characterization and delineation of a new species, *Yersinia wautersii*. Int. J. Med. Microbiol. **304**, 452-463. (10.1016/j.ijmm.2014.02.002) 24598372

[74] Sprague LD, Scholz HC, Amann S, Busse HJ, Neubauer H. 2008 *Yersinia similis* sp. nov. Int. J. Syst. Evol. Microbiol. **58**, 952-958. (10.1099/ijs.0.65417-0) 18398201

[75] Bercovier H, Mollaret HH, Alonso JM, Brault J, Fanning GR, Steigerwalt AG, Brenner DJ. 1980 Intra- and interspecies relatedness of *Yersinia pestis* by DNA hybridization and its relationship to *Yersinia pseudotuberculosis*. Curr. Microbiol. **4**, 225-229. (10.1007/BF02605861)

[76] Gomez-Gil B, Thompson CC, Matsumura Y, Sawabe T, Iida T, Christen R, Thompson F, Sawabe T. 2014 The family *Vibrionaceae*. In The Prokaryotes: Gammaproteobacteria (eds E Rosenberg, EF DeLong, S Lory, E Stackebrandt, F Thompson), pp. 660-747. Berlin, Germany: Springer.

[77] Kilian M, Riley DR, Jensen A, Bruggemann H, Tettelin H. 2014 Parallel evolution of *Streptococcus pneumoniae* and *Streptococcus mitis* to pathogenic and mutualistic lifestyles. MBio **5**, e01490-14. (10.1128/mBio.01490-14) 25053789 PMC4120201

[78] Bishop CJ, Aanensen DM, Jordan GE, Kilian M, Hanage WP, Spratt BG. 2009 Assigning strains to bacterial species via the internet. BMC Biol. **7**, 3. (10.1186/1741-7007-7-3) 19171050 PMC2636762

[79] Didelot X et al. 2011 Recombination and population structure in *Salmonella enterica*. PLoS Pathog. **7**, e1002191. (10.1371/journal.ppat.1002191) 21829375 PMC3145606

[80] den Bakker HC et al. 2011 Genome sequencing reveals diversification of virulence factor content and possible host adaptation in distinct subpopulations of *Salmonella enterica*. BMC Genom. **12**, 425. (10.1186/1471-2164-12-425) PMC3176500 21859443

[81] Timme RE et al. 2013 Phylogenetic diversity of the enteric pathogen *Salmonella enterica* subsp. *enterica* inferred from genome-wide reference-free SNP characters. Genome Biol. Evol. **5**, 2109-2123. (10.1093/gbe/evt159) 24158624 PMC3845640

[82] Parsons SK, Bull CM, Gordon DM. 2011 Substructure within *Salmonella enterica* subspecies *enterica* isolated from Australian wildlife. Appl. Environ. Microbiol. **77**, 3151-3153. (10.1128/AEM.02764-10) 21378038 PMC3126398

[83] Worley J, Meng J, Allard MW, Brown EW, Timme RE. 2018 *Salmonella enterica* phylogeny based on whole-genome sequencing reveals two new clades and novel patterns of horizontally acquired genetic elements. MBio **9**, e02303-18. (10.1128/mBio.02303-18) PMC6282209 30482836

[84] Key FM et al. 2020 Emergence of human-specific *Salmonella enterica* is linked to the Neolithization process. Nature Ecol. Evol. **4**, 324-333. (10.1038/s41559-020-1106-9) 32094538 PMC7186082

[85] Guibourdenche M, Roggentin P, Mikoleit M, Fields PI, Bockemuhl J, Grimont PA, Weill F-X. 2010 Supplement 2003–2007 (no. 47) to the White-Kauffmann-Le Minor scheme. Res. Microbiol. **161**, 26-29. (10.1016/j.resmic.2009.10.002) 19840847

[86] Issenhuth-Jeanjean S, Roggentin P, Mikoleit M, Guibourdenche M, De PE, Nair S, Fields PI, Weill F-X. 2014 Supplement 2008–2010 (no. 48) to the White-Kauffmann-Le Minor scheme. Res. Microbiol. **165**, 526-530. (10.1016/j.resmic.2014.07.004) 25049166

[87] Grimont PA, Weill F-X. 2007 Antigenic formulae of the *Salmonella* serovars, 9th edn. Paris, France: WHO Collaborating Centre for Reference and Research on *Salmonella*.

[88] Seif Y, Monk JM, Machado H, Kavvas E, Palsson BO. 2019 Systems biology and pangenome of *Salmonella* O-Antigens. MBio **10**, e01247-19. (10.1128/mBio.01247-19) 31455646 PMC6712391

[89] Zhang S, den Bakker HC, Li S, Chen J, Dinsmore BA, Lane C, Lauer AC, Fields PI, Deng X. 2019 SeqSero2: rapid and improved *Salmonella* serotype determination using whole genome sequencing data. Appl. Environ. Microbiol. **85**, e01746-19. (10.1128/AEM.01746-19) 31540993 PMC6856333

[90] Robertson J, Yoshida C, Kruczkiewicz P, Nadon C, Nichani A, Taboada EN, Nash JHE. 2018 Comprehensive assessment of the quality of *Salmonella* whole genome sequence data available in public sequence databases using the *Salmonella* *in silico* Typing Resource (SISTR). Microb. Genom. **4**, 1-11. (10.1099/mgen.0.000151) PMC5857378 29338812

[91] Uelze L, Borowiak M, Deneke C, Szabo I, Fischer J, Tausch SH, Malorny B. 2020 Performance and accuracy of four open-source tools for *in silico* serotyping of *Salmonella* spp. based on whole-genome short-read sequencing data. Appl. Environ. Microbiol. **86**, e02265-19. (10.1128/AEM.02265-19) 31862714 PMC7028957

[92] Gladstone RA et al. 2019 International genomic definition of pneumococcal lineages, to contextualise disease, antibiotic resistance and vaccine impact. EBioMed. **43**, 338-346. (10.1016/j.ebiom.2019.04.021) PMC6557916 31003929

[93] Pedregosa F et al. 2011 Scikit-learn: machine learning in Python. J. Machine Learn. Res. **12**, 2825-2830.

[94] Romano S, Vinh NX, Bailey J, Verspoor K. 2016 Adjusting for chance clustering comparison measures. J. Machine Learn. Res. **17**, 1-32.

[95] Bostock M, Ogievetsky V, Heer J. 2011 D^3^: Data-Driven Documents. IEEE Trans. Vis. Comput. Graph **17**, 2301-2309. (10.1109/TVCG.2011.185) 22034350

[96] Chaudhuri RR, Henderson IR. 2012 The evolution of the *Escherichia coli* phylogeny. Infect. Genet. Evol. **12**, 214-226. (10.1016/j.meegid.2012.01.005) 22266241

[97] Ochman H, Selander RK. 1984 Standard reference strains of *Escherichia coli* from natural populations. J. Bacteriol. **157**, 690-693. (10.1128/jb.157.2.690-693.1984) 6363394 PMC215307

[98] Selander RK, Caugant DA, Whittam TS. 1987 Genetic structure and variation in natural populations of *Escherichia coli*. In *Escherichia coli* and *Salmonella typhimurium* cellular and molecular biology, vol. II (eds FC Neidhardt, JL Ingraham, KB Low, B Magasanik, M Schaechter, HE Umbarger), pp. 1625-1648. Washington, DC: American Society for Microbiology.

[99] Touchon M, Perrin A, de Sousa JAM, Vangchhia B, Burn S, O'Brien CL, Denamur E, Gordon D, Rocha EP. 2020 Phylogenetic background and habitat drive the genetic diversification of *Escherichia coli*. PLoS Genet. **16**, e1008866. (10.1371/journal.pgen.1008866) 32530914 PMC7314097

[100] Denamur E, Clermont O, Bonacorsi S, Gordon D. 2021 The population genetics of pathogenic *Escherichia coli*. Nat. Rev. Microbiol. **19**, 37-54. (10.1038/s41579-020-0416-x) 32826992

[101] Gordon DM, Clermont O, Tolley H, Denamur E. 2008 Assigning *Escherichia coli* strains to phylogenetic groups: multi-locus sequence typing versus the PCR triplex method. Environ. Microbiol. **10**, 2484-2496. (10.1111/j.1462-2920.2008.01669.x) 18518895

[102] Clermont O, Christenson JK, Denamur E, Gordon DM. 2013 The Clermont *Escherichia coli* phylo-typing method revisited: improvement of specificity and detection of new phylo-groups. Environ. Microbiol. Rep. **5**, 58-65. (10.1111/1758-2229.12019) 23757131

[103] Beghain J, Bridier-Nahmias A, Le NH, Denamur E, Clermont O. 2018 ClermonTyping: an easy-to-use and accurate *in silico* method for *Escherichia* genus strain phylotyping. Microb. Genom. **4**, e000192. (10.1099/mgen.0.000192) PMC6113867 29916797

[104] Waters NR, Abram F, Brennan F, Holmes A, Pritchard L. 2020 Easy phylotyping of *Escherichia coli* via the EzClermont web app and command-line tool. Access Microbiol. **2**, 1-5. (10.1099/acmi.0.000143) PMC7656184 33195978

[105] Sawyer C et al. 2021 Epidemiological investigation of recurrent outbreaks of haemolytic uraemic syndrome caused by Shiga toxin-producing *Escherichia coli* serotype O55:H7 in England, 2014–2018. Epidemiol. Infect. **149**, e108. (10.1017/S0950268821000844) 33866980 PMC8161408

[106] Wick LM, Qi W, Lacher DW, Whittam TS. 2005 Evolution of genomic content in the stepwise emergence of *Escherichia coli* O157:H7. J. Bacteriol. **187**, 1783-1791. (10.1128/JB.187.5.1783-1791.2005) 15716450 PMC1064018

[107] Eichhorn I, Semmler T, Mellmann A, Pickard D, Anjum MF, Fruth A, Karch H, Wieler LH. 2018 Microevolution of epidemiological highly relevant non-O157 enterohemorrhagic *Escherichia coli* of serogroups O26 and O111. Int. J. Med. Microbiol. **308**, 1085-1095. (10.1016/j.ijmm.2018.08.003) 30115547

[108] Liu CM et al. 2018 *Escherichia coli* ST131-H22 as a foodborne uropathogen. MBio **9**, e00470-18. (10.1128/mBio.00470-18) 30154256 PMC6113624

[109] Price LB et al. 2013 The epidemic of extended-spectrum-beta-lactamase-producing *Escherichia coli* ST131 is driven by a single highly pathogenic subclone, H30-Rx. MBio **4**, e00377-13. (10.1128/mBio.00377-13) 24345742 PMC3870262

[110] Achtman M, Mercer A, Kusecek B, Pohl A, Heuzenroeder M, Aaronson W, Sutton A, Silver RP. 1983 Six widespread bacterial clones among *Escherichia coli* K1 isolates. Infect. Immun. **39**, 315-335. (10.1128/iai.39.1.315-335.1983) 6218094 PMC347943

[111] Gordon DM et al. 2017 Fine-scale structure analysis shows epidemic patterns of Clonal Complex 95, a cosmopolitan *Escherichia coli* lineage responsible for extraintestinal infection. mSphere **2**, e00168-17. (10.1128/mSphere.00168-17) 28593194 PMC5451516

[112] Frentrup M et al. 2020 A publicly accessible database for *Clostridioides difficile* genome sequences supports tracing of transmission chains and epidemics. Microbial Genom. **6**, mgen.0.000410. (10.1099/mgen.0.000410) PMC7641423 32726198

[113] Laukkanen-Ninios R et al. 2011 Population structure of the *Yersinia pseudotuberculosis* complex according to multilocus sequence typing. Environ. Microbiol. **13**, 3114-3127. (10.1111/j.1462-2920.2011.02588.x) 21951486 PMC3988354

[114] Lees JA, Harris SR, Tonkin-Hill G, Gladstone RA, Lo SW, Weiser JN, Corander J, Bentley SD, Croucher NJ. 2019 Fast and flexible bacterial genomic epidemiology with PopPUNK. Genome Res. **29**, 304-316. (10.1101/gr.241455.118) 30679308 PMC6360808

[115] Lees JA, Tonkin-Hill G, Yang Z, Corander J. 2022 Mandrake: visualising microbial population structure by embedding millions of genomes into a low-dimensional representation. *Phil. Trans. R. Soc. B* **377**, 20210237. (10.1098/rstb.2021.0237) PMC9393562 35989601

[116] Tschoeke D, Salazar VW, Vidal L, Campeao M, Swings J, Thompson F, Thompson C. 2020 Unlocking the genomic taxonomy of the *Prochlorococcus* collective. Microb. Ecol. **80**, 546-558. (10.1007/s00248-020-01526-5) 32468160

[117] Salazar VW, Tschoeke DA, Swings J, Cosenza CA, Mattoso M, Thompson CC, Thompson FL. 2020 A new genomic taxonomy system for the *Synechococcus* collective. Environ. Microbiol. **22**, 4557-4570. (10.1111/1462-2920.15173) 32700350

[118] Achtman M, Pluschke G. 1986 Clonal analysis of descent and virulence among selected *Escherichia coli*. Annu. Rev. Microbiol. **40**, 185-210. (10.1146/annurev.mi.40.100186.001153) 3535645

[119] Carroll KJ, Jenkins C, Harvey-Vince L, Mohan K, Balasegaram S. 2021 Shiga toxin-producing *Escherichia coli* diagnosed by Stx PCR: assessing the public health risk of non-O157 strains. Eur. J. Public Health **31**, 576-582. (10.1093/eurpub/ckaa232) 33411922

[120] Achtman M, Zhou Z, Charlesworth J, Baxter L. 2022 EnteroBase: hierarchical clustering of 100 000s of bacterial genomes into species/subspecies and populations. Figshare. (10.6084/m9.figshare.c.6097222) PMC9393565 35989609

